# Proangiogenic Properties of Extracellular Vesicles Secreted by Endothelial Cells Reversibly Primed for Anoikis: A Possible Autocrine Mechanism Induced by Astrocytoma Extracellular Matrix

**DOI:** 10.3390/ijms27062574

**Published:** 2026-03-11

**Authors:** Aline O. da Silva-de-Barros, Tercia Rodrigues Alves, Laila Ribeiro-Fernandes, Edward Helal-Neto, Ana Clara Frony, Bruno Pontes, Nathan Bessa Viana, Paula Kubitschek Barreira, Nathália Curty, Andrés Rodríguez-Vega, Carla-Verônica Loureiro y Penha, João Alfredo de Moraes, Vivaldo Moura-Neto, Christina Barja-Fidalgo, Verônica Morandi

**Affiliations:** 1 Laboratório de Biologia da Célula Endotelial & Angiogênese (LabAngio), Departamento de Biologia Celular, Instituto de Biologia Roberto Alcantara Gomes (IBRAG), Universidade do Estado do Rio de Janeiro (UERJ), Campus Maracanã, Rio de Janeiro 20550-013, RJ, Brazil; alinedbcg@gmail.com (A.O.d.S.-d.-B.); terciabio@yahoo.com.br (T.R.A.); lailarfernandes@gmail.com (L.R.-F.); edwneto@gmail.com (E.H.-N.); 2 Faculdade de Ciências Biológicas e Saúde, Universidade do Estado do Rio de Janeiro (UERJ), Campus Zona Oeste, Rio de Janeiro 23070-200, RJ, Brazil; 3 Laboratório de Farmacologia Celular & Molecular (LFCM), IBRAG, Departamento de Biologia Celular, Universidade do Estado do Rio de Janeiro (UERJ), Campus de Ciências Biológicas e da Saúde I, Vila Isabel, Rio de Janeiro 20550-170, RJ, Brazil; clarafrony@gmail.com (A.C.F.); barja-fidalgo@uerj.br (C.B.-F.); 4 Instituto de Ciências Biomédicas, Universidade Federal do Rio de Janeiro (UFRJ), Cidade Universitária, Ilha do Fundão, Rio de Janeiro 21941-902, RJ, Brazil; bpontes@icb.ufrj.br (B.P.); joaomoraes@icb.ufrj.br (J.A.d.M.); 5 Centro Nacional de Biologia Estrutural e Bioimagem (CENABIO), Universidade Federal do Rio de Janeiro (UFRJ), Cidade Universitária, Ilha do Fundão, Rio de Janeiro 21941-902, RJ, Brazil; nathan@if.ufrj.br; 6 Instituto de Física, Universidade Federal do Rio de Janeiro (UFRJ), Cidade Universitária, Ilha do Fundão, Rio de Janeiro 21941-909, RJ, Brazil; 7 Centro de Pesquisa em Biologia Celular & Ômicas (BIO-OMICS), Instituto de Biologia Roberto Alcantara Gomes, Departamento de Biologia Celular, Universidade do Estado do Rio de Janeiro (UERJ), Campus Maracanã, Rio de Janeiro 20550-013, RJ, Brazil; paulahkb@gmail.com (P.K.B.); ncurtydeandrade@gmail.com (N.C.); andresve22@gmail.com (A.R.-V.); carlapenha@hotmail.com (C.-V.L.y.P.); 8 Instituto Estadual do Cérebro Paulo Niemeyer (IEC), Centro, Rio de Janeiro 20230-025, RJ, Brazil; vivaldomouraneto@gmail.com

**Keywords:** angiogenesis, glioblastoma, extracellular matrix, extracellular vesicles, tenascin-C, tubulogenesis, apoptosis, anoikis, cell migration, glycolysis

## Abstract

Altered extracellular matrix (ECM), a hallmark of solid tumors, affects cellular survival, migration and differentiation. Typically viewed as tumor-suppressive, evidence suggests that apoptosis can also generate pro-tumoral signals. We previously showed that ECM from high-grade astrocytomas induces extensive endothelial anoikis, while a surviving subpopulation fails to form tubular structures (tubulogenesis-defective endothelial cells, or TDECs). We combined functional assays with whole-cell proteomics to investigate this response. Using real-time video microscopy, we found that apoptotic endothelial cells induced by tumor ECM attracted migrating endothelial cells and guided sprouting. Conditioned media from apoptotic endothelial cells contained a 2.8-fold increase in extracellular vesicles (EVs) relative to autologous ECM-primed endothelial cells. Although both EV populations improved TDEC tubulogenesis, only EVs produced upon tumor-ECM stimulation induced TDEC migration—a property lost when using EVs secreted by endothelial cells growing on TN-C-depleted matrices. Proteomic profiling revealed that TDECs shift from an adhesion-anchored to a microtubule-rich and glycolytically rewired phenotype, with upregulation of vesicle-trafficking regulators (ARF1/3/4, ANXA2/5), migration drivers (RAC1/3, RHOA/C, WDR1, FSCN1) and glycolytic enzymes (ENO1, ALDOA, PKM, LDHA), alongside the suppression of integrin- and cytoskeletal-anchoring proteins. Collectively, these findings indicate that tumor-ECM-driven endothelial apoptosis generates reversible reprogramming and an EV-mediated autocrine mechanism that may favor angiogenic balance.

## 1. Introduction

Cancer progression is dependent on abnormal angiogenesis that supplies oxygen, growth factors, and nutrients to facilitate tumor growth and metastasis progression [1]. High-grade gliomas are the most common brain tumors, and exuberant aberrant angiogenesis is a hallmark of aggressive glioblastoma (GBM), remaining a crucial therapeutic target in its treatment. Despite intensive research, GBMs still present a poor prognosis [2,3,4]. These tumors can modify their stromal environment, altering the behavior of neighboring normal cells, including endothelial cells [5,6].

Recent studies have shown that the microenvironment of most tumors can increase the secretion of extracellular vesicles (EVs), which can fuse with cell membranes, thus allowing communication between tumors and surrounding cells [7]. EVs are defined as a set of secreted or shed membrane vesicles that include exosomes, microvesicles, and apoptotic bodies. Exosomes are 40–150 nm (average~100 nm) vesicles generated by exocytosis from multivesicular bodies (MVBs) derived from the endocytic pathway and secreted when these compartments fuse with the plasma membrane [8]. Microvesicles are shed from the plasma membrane via direct outward budding and range in size from 50–1000 nm. In contrast, apoptotic bodies are released during apoptosis and typically measure 1000–5000 nm [9,10]. EVs can carry a broad repertoire of cargoes, including cytokines, membrane receptors and receptor ligands, lipids, and nucleic acids [11,12]. Tumor-derived EVs can affect angiogenesis by modulating endothelial cell proliferation, migration, and adhesion, as well as by influencing the production of pro- and antiangiogenic factors [13,14,15,16].

In addition, accumulating evidence indicates that endothelial cells develop a vesicular network that also plays a vital role in EV biogenesis and uptake [16,17]. After engaging with the endothelial surface, extracellular vesicles can enter cells via well-established endocytic pathways, including macropinocytosis, clathrin-mediated endocytosis, and caveolae-mediated endocytosis [18]. The release of EVs by endothelial cells has primarily been shown to be induced by soluble mediators, especially during inflammation. Key mediators include interleukin-1 (IL-1), interferon-γ (IFN-γ), and bacterial lipopolysaccharide (LPS) [17]. Additionally, C-reactive protein (CRP) and plasminogen activator inhibitor-1 (PAI-1) have also been implicated in promoting the release of EVs from endothelial cells [19]. Although empirical evidence is scarce, endothelial cell-derived EVs have been shown to autocrinally modulate angiogenic properties within the tumor microenvironment in response to inflammatory stimuli [16].

Apoptosis is commonly observed in the low-oxygen microenvironments of advanced solid tumors [20,21,22], where tumor cells extensively remodel the extracellular matrix (ECM), which plays a pivotal role in tumor growth and angiogenesis [23]. Apoptosis has long been recognized as an essential component of physiological angiogenesis, particularly in vessel pruning and regression [24,25,26,27].

We have previously shown that immobilized ECMs secreted by high-grade astrocytoma cell lines induced a gradual endothelial cell apoptosis by anoikis (40–80% cell death along the first 24 h of incubation with astrocytoma ECM) compared with non-tumoral ECMs [28]. Importantly, the remaining adherent endothelial subpopulation became defective in forming organized tubular structures, a defect associated with the elevated tenascin-C-to-fibronectin (TN:FN) ratio observed in tumoral matrices [28]. The tubulogenic defect was reversed when the immobilized tumor matrix was pre-treated with anti-tenascin-C neutralizing antibodies, indicating that the adherent endothelial population had been primed—but not irreversibly committed—to anoikis. However, this study did not investigate whether apoptotic detached cells and their putative EVs could, in an autocrine manner, influence the morphological differentiation of the ‘anoikis-primed’ but still fully viable endothelial subpopulation. Furthermore, published studies directly identifying tumor ECM as the primary trigger of endothelial EV production remain exceedingly scarce.

Since the two endothelial cell subpopulations—anoikis-sensitive and viable adherent cells—would be expected to coexist in the same TN-C-rich microenvironment as the tumor progresses, our goal in the present work was to investigate how endothelial apoptosis, explicitly triggered by the astrocytoma ECM, influences the angiogenic behavior of viable endothelial cells primed by the same ECM. We found a role of apoptotic cells and their related EVs in several angiogenic events, including endothelial tubulogenesis, branching, and cell migration.

## 2. Results

### 2.1. Apoptotic Endothelial Cell-Oriented Attraction of Endothelial Cells

**Figure 1:**
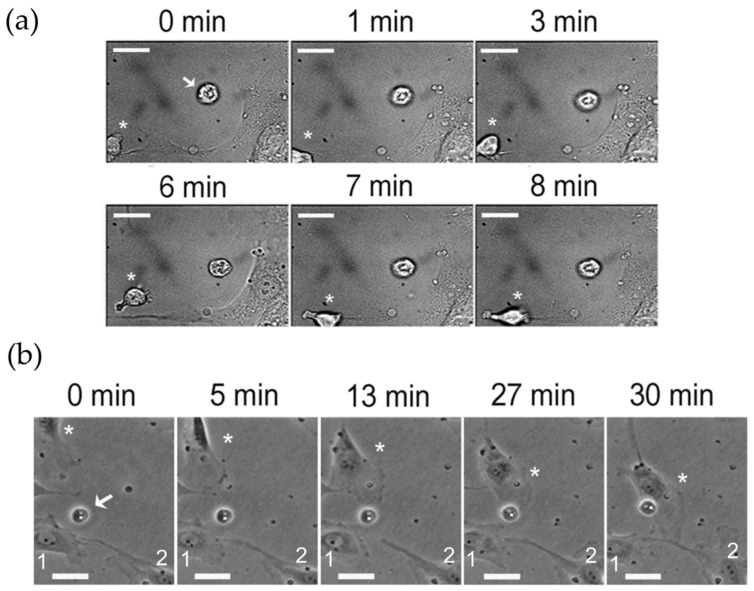
Endothelial cell attraction by endothelial apoptotic cells. Apoptotic cells were generated by anoikis upon the prolonged incubation of HUVECs with immobilized U251-MG ECM, as described in the Section 4. Using optical tweezers and time-lapse video microscopy, we successfully positioned single apoptotic cells (indicated by arrows) near viable endothelial cells (indicated by asterisks or numbers). In (**a**), an apoptotic endothelial cell is positioned near the endothelial monolayer (arrow), with a migratory ameboid endothelial cell exhibiting directed membrane extensions (t = 6 min) and filopodia (t = 7–8 min). In a longer recording series (**b**), a cell (asterisk) displaying a mesenchymal-like migratory phenotype appears to establish cytoplasmic connections with other adherent cells (indicated by the numbers) after first moving to and contacting the dead cell (t = 27 min, arrow) that had been displaced to the vicinity of the stationary cells. Stationary cell #1 also extends membrane ruffles toward the dead cell (t = 5–13 min) and seems to embrace it. Bars represent 20 μm.

Weihua and coworkers [24] demonstrated that within a 200-µm radius, apoptotic endothelial cells can induce sprout formation. Here, we tested whether endothelial cells rendered apoptotic by anoikis after incubation with astrocytoma ECM could induce the migration of healthy endothelial cells in vitro. We added apoptotic endothelial cells to the culture and performed time-lapse video microscopy. Using optical tweezers, we were able to position single apoptotic cells near viable endothelial cells (cultivated under standard conditions) at distances < 100 µm. Figure 1 shows a representative record using this approach: in the first register (t = 0–8 min), we observed an endothelial cell exhibiting an ameboid-like migratory phenotype, apparently extending filopodia toward a manipulated apoptotic cell at t = 6 min (Figure 1a). In a longer recording (t = 5 to 30 min), we registered images of a mesenchymal-like migratory endothelial cell that seemingly was attracted by the apoptotic cell that had been positioned with the optical tweezers and then established new contacts with two other endothelial cells adjacent to the place of manipulation (Figure 1b).

These images were highly suggestive of the role of apoptotic cells induced by astrocytoma ECM in rapidly driving the directional movement of endothelial cells in their near microenvironment.

### 2.2. Apoptotic Endothelial Cells Guide the Formation of Endothelial Sprouts In Vitro

**Figure 2:**
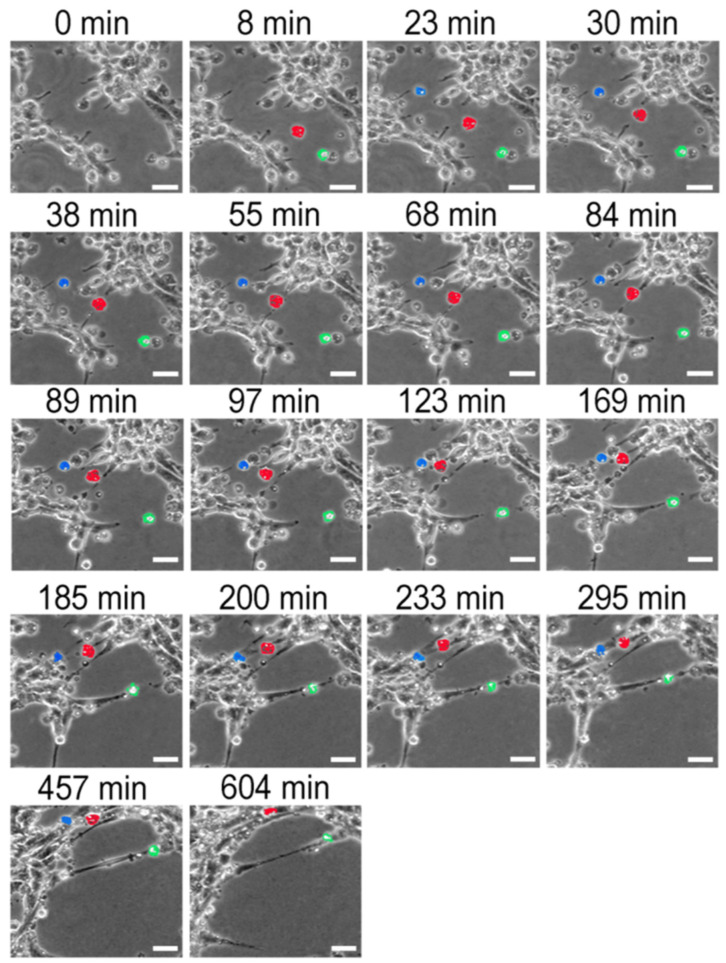
Apoptotic endothelial cells induce endothelial sprouting. Endothelial cells rendered apoptotic by anoikis after incubation with immobilized matrix secreted by U251-MG cells or by healthy HUVECs were used in this experiment. Using time-lapse video microscopy, it was possible to track apoptotic cell-guided sprouting from healthy endothelial cells grown on Matrigel™ pellets. In these images, the digitally colored red apoptotic cell had linked two nascent sprouts by 40 min into the assay. These sprouts joined together after the displacement of apoptotic red cells (89–169 min). The red cell initiates the same process at another forming sprout (123–233 min). By 457–604 min, it exhibited typical apoptotic shrinkage and a fragmented appearance. Similar sprout-like structures were observed in blue- and green-stained apoptotic endothelial cells. Bars represent 20 μm.

To further investigate whether apoptotic cells affect the ability of viable endothelial cells to establish network-like connections, thereby possibly modulating their capacity to form tube-like structures, we combined time-lapse video microscopy with a 3D Matrigel™ assay (Figure 2). To better track apoptotic cells, they were digitally labeled in blue, red, and green (the figure showing the untagged apoptotic endothelial cells is available; see Figure S1, Supplementary Materials). Four hours after seeding cells on Matrigel, when some short initial sprouts were spotted, apoptotic endothelial cells were added to the Matrigel pellets (t = 0 min). Interestingly, after 23 min, when all the added endothelial apoptotic cells had reached the Matrigel™ pellet, some of them appeared to be positioned between the sprouts in formation (Figure 2, 23–200 min). The subsequent images suggest their participation in a branching organization, as they connected two opposite sprouts (details of this analysis are provided in the legend for Figure 2).

### 2.3. Apoptotic Endothelial Cells Stimulate Tubulogenesis In Vitro in a Density-Dependent Manner

**Figure 3:**
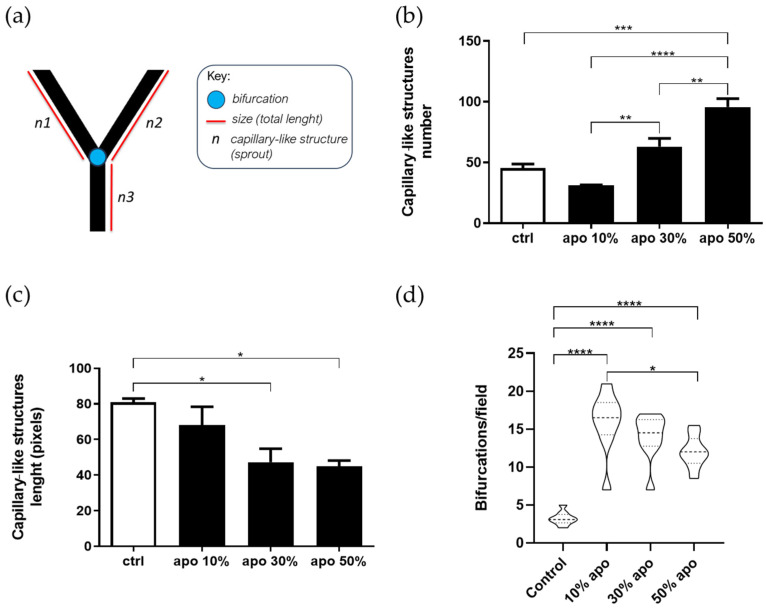
Apoptotic endothelial cells improve angiogenic differentiation in tubulogenesis-defective endothelial cells (TDECs). TDECs were prepared by seeding HUVECs on the immobilized extracellular matrix secreted by U251-MG cells, as described in the Section 4, and then subjected to the Matrigel™ assay in the presence of the indicated amounts of apoptotic endothelial cells (apo) suspended in M199 supplemented with 5% FCS. After 16 h, the samples were fixed in 1.1% glutaraldehyde in PBS. (**a**) Explanation of the parameters used to quantify angiogenic differentiation; (**b**) Total number of capillary-like structures; (**c**) Total length of structures; and (**d**) Total number of endothelial bifurcations. Two independent observers performed the count. In each experiment, determinations were performed in quadruplicate for each condition. Data are presented as mean ± SD from 3 independent experiments. * *p* < 0.05; ** *p* < 0.01; *** *p* < 0.001, **** *p* < 0.0001.

As we have previously demonstrated, the TN-C–rich extracellular matrix secreted by U251-MG astrocytoma cells selectively gives rise to two endothelial cell subpopulations: one that becomes defective in tubulogenesis and another that undergoes detachment-induced apoptosis, as confirmed by routine annexin V/PI double staining and the TUNEL assay [28]. Since we observed that connections between sprouting endothelial cells could be stimulated by the presence of the dead endothelial cells that underwent anoikis (induced by the ECM secreted by astrocytoma cells), we decided to quantify the formation of tube-like structures but now using these tubulogenesis-defective endothelial cells (TDECs) when they were exposed to increasing amounts of apoptotic cells by performing a long-term (16–25 h) tridimensional tubulogenesis assay (Figure 3).

Endothelial apoptotic cells were expressed as a percentage of total TDECs seeded on Matrigel™ pellets. The following parameters of endothelial differentiation were evaluated (Figure 3a): (i) number of tube-like structures (sprouts), (ii) total tube length, and (iii) number of bifurcations. A significant increase in the number of tube-like structures was observed at dead-to-TDEC ratios of 1:3–1:2 (Figure 3b and Figure S2 for a morphological record, Supplementary Material file), although the total tube length decreased under the same conditions (Figure 3c). The number of bifurcations, a key indicator of network branching that correlates with tip-cell sprouting [29], increased at all apoptotic cell ratios tested (10–50%), with at least a fourfold rise relative to the control (Figure 3d). The most pronounced effect occurred at the lowest apoptotic cell density (10%).

### 2.4. Endothelial Anoikis Induced by Astrocytoma ECM Promotes the Shedding of Extracellular Vesicles (EVs)

Over the last few years, the relationship between apoptosis and extracellular vesicle (EV) biogenesis has become increasingly evident [20,21,30]. We have previously shown that endothelial cell death by anoikis was consistently induced by the ECM produced by either established astrocytoma cell lines (human U251-MG, and U87; rat C6 cells) or by primary-derived cells grown from human astrocytoma explants (GBM02, GBM03, GBM95) [28]. Nevertheless, the impact of EVs on angiogenesis in the context of endothelial anoikis induced by the matrix of these clinically relevant tumors has not been reported in the literature to date.

**Figure 4:**
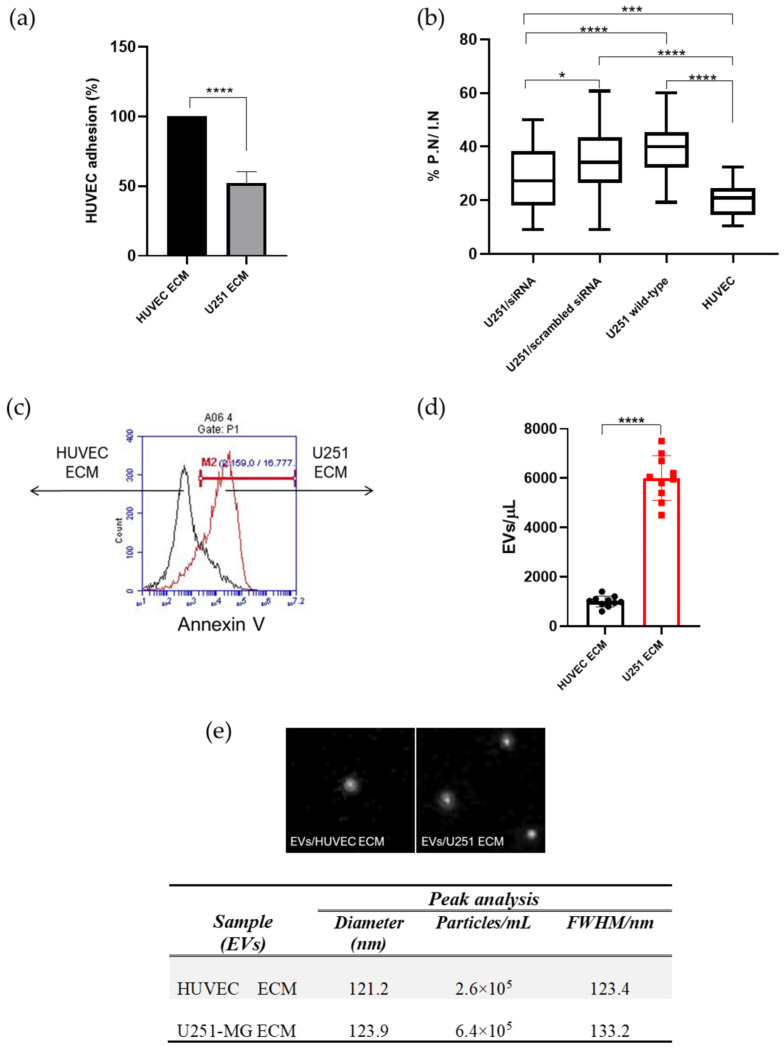
Quantification and size characterization of extracellular vesicles (EVs) isolated from endothelial conditioned media. (**a**) Adhesion rates of HUVECs seeded onto either autologous extracellular matrix (ECM) or ECM secreted by U251-MG astrocytoma cells (n = 5). (**b**) Unfractionated conditioned media from both conditions, containing detached endothelial cells with either pyknotic or intact nuclei, were cleared of cells and large debris and subjected to EV isolation by ultracentrifugation (n = 3). (**c**,**d**) EV abundance was estimated by flow cytometry based on the detection of annexin V-positive vesicles with diameters < 1 µm. (**c**) A representative cytometry histogram of EVs isolated from endothelial cells growing on autologous matrix (control, black) or on tumor matrix (red); (**d**) Quantification of Annexin V–positive EVs across independent experiments (ten EVs preparations from three independent experiments). (**e**) Representative nanoparticle tracking analysis (NTA) from five independent EV preparations showing peak analysis, mean particle diameters, and size distribution. Vesicles larger than ~500 nm in diameter were not detected in any condition (see also Figure S5 for additional information). PN: cells bearing pyknotic nuclei; IN: cells bearing intact nuclei; n = number of independent experiments; Data are presented as mean ± SD from independent experiments, as indicated for each panel. * *p* < 0.05; *** *p* < 0.001; **** *p* < 0.0001.

Thus, we decided to elucidate and characterize the presence of EVs in the conditioned medium of endothelial cells rendered apoptotic after 24 h of incubation with ECM secreted by U251-MG astrocytoma cells compared with the conditioned medium of endothelial cells incubated with their own immobilized ECM (autologous matrix) (Figure 4). To ensure that EVs were isolated from endothelial cells undergoing anoikis, we quantified cell adhesion in all samples after 24 h of incubation, as well as the number of cells bearing pyknotic nuclei, a hallmark of advanced apoptosis (Figure 4a,b; Figure S3, for morphological representations of pyknotic nuclei used in the quantifications).

Endothelial detachment induced by U251 ECM was at least ≈60% greater than observed in endothelial cells incubated with their autologous matrix (Figure 4a). We previously demonstrated that under the conditions of this attachment assay, the ratio of pyknotic nuclei to intact nuclei (PN:IN) constitutes a bona fide indicator of apoptosis, as independently validated by annexin-V/PI staining and TUNEL analysis [28]. Consistent with this, endothelial cells seeded on glioma-derived matrix displayed a 93% increase in the PN:IN ratio compared with cells seeded on autologous matrix (Figure 4b). Silencing TN-C (at levels above 90%) in U251-MG cells before matrix deposition decreased the number of endothelial pyknotic nuclei by 75% (U251/siRNA vs. U251/scrambled siRNA), supporting the key role of this matricellular protein in the selection of endothelial sub phenotypes with distinct angiogenic capacities [28] (Figure 4b; Figure S4, for TN-C silencing evidence by western blotting, Supplementary Materials).

After confirming the apoptotic nature of the starting material, conditioned media containing dead endothelial cells and their cellular fragments—along with the expected range of smaller particles and vesicles released from viable or apoptotic cells—were fractionated and purified by ultracentrifugation. The samples were then analyzed by flow cytometry to detect extracellular vesicles (EVs) with diameters up to 1 µm. Incubation of endothelial cells with an astrocytoma-derived matrix resulted in a 2.8-fold increase in EV release compared with cells maintained on their autologous matrix (Figure 4c,d).

Quantification by nanoparticle tracking analysis (NTA) (Figure 4e) corroborated the cytometric analysis, showing a 2.46-fold increase in EVs secreted by TDECs. Size distribution analysis by NTA showed that both conditions generated EVs with comparable mean diameters (121.2 and 123.9 nm for HUVEC ECM and U251 ECM, respectively) and similar distribution widths (FWHM = 123.4 and 133.2 nm for the same conditions, respectively). Only residual particles outside the main peaks displayed maximal diameters of ~500 nm, thereby excluding large apoptotic bodies in our EV preparations (Figure S5a,b, Supplementary Materials).

Furthermore, EVs isolated from both experimental conditions were enriched for the exosomal markers CD63 and annexin A2 when equal amounts of total protein were analyzed, supporting the comparable molecular identity of the EVs preparations (Figure S5c,d, Supplementary Materials).

### 2.5. EVs Derived from Astrocytoma-Induced Endothelial Cells by Anoikis Support Endothelial Tubulogenesis and Cell Migration

**Figure 5:**
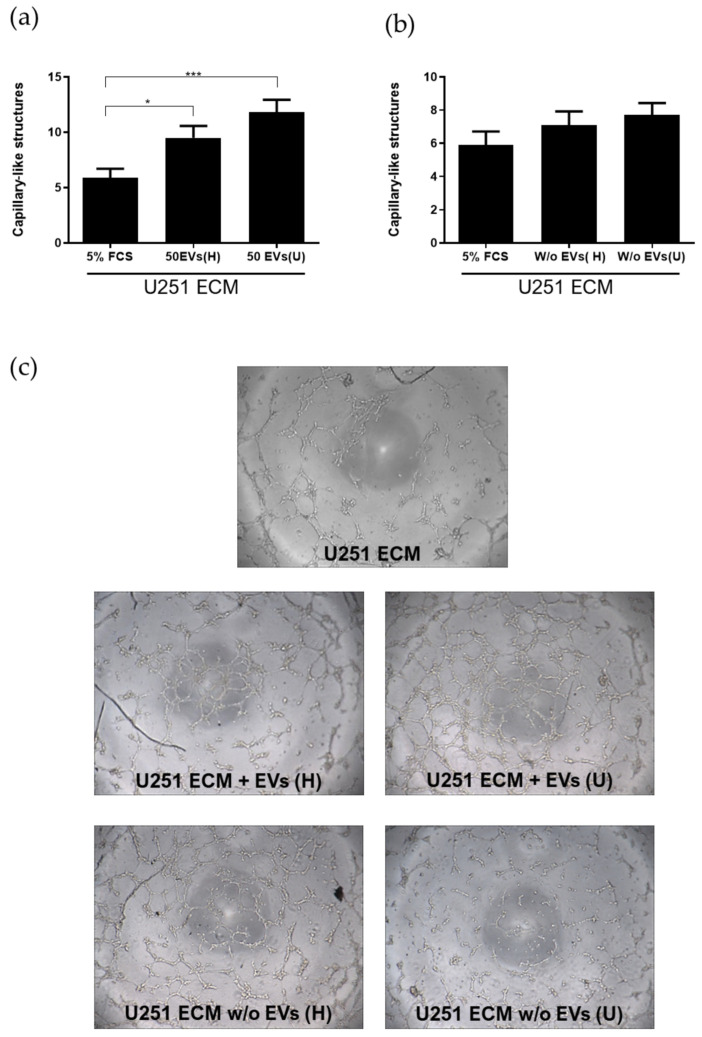
Stimulation of tubulogenesis of TDECs by endothelial EVs. EVs were isolated either from HUVECs detached from their autologous matrix (H) or from the U251-MG matrix (U). (**a**) Tubulogenesis-defective endothelial cells (TDECs) were prepared by seeding HUVECs on immobilized extracellular matrix secreted by U251-MG astrocytoma cells, as described in Section 4, and then submitted to the Matrigel™ assay in the presence of the indicated amounts of EVs, suspended in M199 supplemented with 5% FCS. After 16 h, cells were fixed in 1.1% glutaraldehyde in PBS, and the total number of tube-like structures/well was counted. In (**b**), the same approach was performed after the depletion of EVs from the incubation media. (**c**) Morphological aspect of TDECs on Matrigel pellets after 16 h. Data are presented as mean ± SD from three independent experiments; * *p* < 0.05; *** *p* < 0.001.

Since we demonstrated that the endothelial anoikis process induced by a TN-C-rich tumor matrix is accompanied by massive EV production by endothelial cells, we asked whether the stimulation of endothelial migration and tubulogenesis by whole apoptotic endothelial cells and their fragments, observed in later experiments (Figure 1, Figure 2 and Figure 3), could be related to EV generation by apoptotic cells (Figure 5). Using the Matrigel™ tubulogenesis assay again, we found that endothelial cell-derived EVs produced by cells seeded onto the astrocytoma matrix significantly increased TDECs tubulogenesis (Figure 5a,c). Based on pilot experiments, we selected a concentration of 50 EVs/µL, as no measurable effects were observed at lower concentrations. EV levels were estimated from annexin V-positive events using flow cytometry. Concentrations within this range have been used in prior studies reporting functional EV activity [31,32]. The depletion of EVs by ultracentrifugation completely abrogates their positive effect on tubulogenesis (Figure 5b,c). Nevertheless, this induction was not significantly different from that obtained with stimulation using EVs isolated from endothelial cells that had been previously incubated with the autologous matrix (i.e., deposited by healthy endothelial cells, not primed for apoptosis).

**Figure 6:**
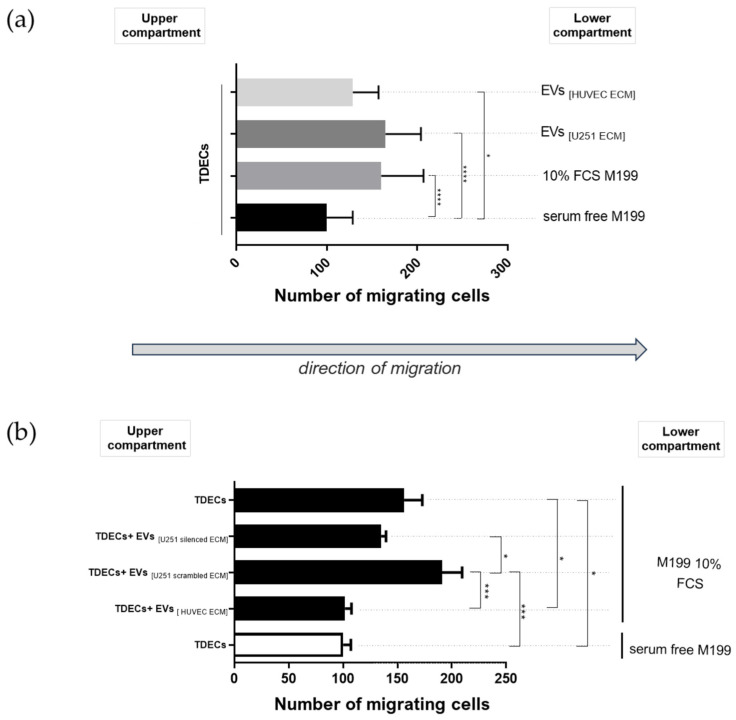
EVs isolated from endothelial cells rendered apoptotic by anoikis induce TDEC chemotaxis. TDECs were prepared by seeding HUVECs on immobilized extracellular matrix secreted by U251-MG astrocytoma cells and then submitted to a chemotaxis assay in a modified Boyden chamber, as described in Section 4. EVs (50 EVs/µL) isolated from HUVECs incubated either with their autologous matrix (EVs_[HUVEC ECM]_) or with tumor matrix (EVs_[U251 ECM]_) were added either to the upper (**a**) or lower (**b**) compartments of the system, as indicated in the panels. U251-MG cells had their TN-C expression manipulated previously to matrix deposition, also to generate EVs_[U251 silenced ECM]_ (EVs isolated from endothelial cells incubated with TN-C-depleted tumor matrix) and EVs_[U251 scrambled ECM]_ (control condition for TN-C silencing). Cells were allowed to migrate for 4 h. The number of cells migrating toward serum-free 199 was set as the baseline for migration (considered as 100%). Data are presented as mean ± SD from three independent experiments in both panels. * *p* < 0.05, *** *p* < 0.001, **** *p* < 0.0001.

At first glance, these results suggest that when both sources of EVs (derived from endothelial cells incubated with either autologous or tumor matrices) were tested at the same concentration, no qualitative differences were observed. However, when evaluated in a functional chemotaxis assay, the two EV populations displayed markedly distinct effects on TDEC behavior (Figure 6). We first examined the migration of TDECs toward different endothelial EVs, which were used as a chemoattractant in the lower compartment of Boyden chambers (Figure 6a). Although a trend toward increased TDEC migration was observed in response to EVs produced by endothelial cells incubated with the tumor-derived matrix, the difference was not statistically significant.

Since physical contact between cells and EVs may involve direct binding and uptake of the vesicles by target cells, thereby ensuring the transfer of signaling and effector molecules [33], we modified the design of the chemotaxis assay to promote close vesicle–TDEC contact within the upper compartment of the Boyden chamber before migration (Figure 6b). TDEC migration was significantly inhibited when these cells were in direct physical contact with EVs produced by healthy endothelial cells (primed for 24 h with their own autologous matrix) before migrating to the lower compartment under FCS stimulation.

Moreover, silencing TN-C expression in tumor cells before the deposition of the immobilized matrix led to the generation of endothelial cell-derived EVs that elicited markedly reduced TDEC migration when incubated with the cells in the upper compartment of the Boyden chamber, compared with the TN-C-silencing control condition (U251-silenced ECM vs. U251-scrambled ECM, Figure 6b). These findings indicate that EVs isolated from endothelial cells incubated with the tumor matrix differ from those secreted by healthy endothelial cells, not only in quantity but also in quality, potentially influencing angiogenesis.

### 2.6. Whole-Cell Proteomic Profiling of TDECs Reveals Cell-Matrix-Driven Rewiring of Endothelial Machinery

We previously reported that endothelial cells initially adhere to and spread on glioma-derived extracellular matrix (ECM) from different astrocytoma sources. Still, after 4–6 h of adhesion, they gradually initiate detachment and undergo anoikis over the next several hours—a selective effect not observed in normal human fibroblasts exposed to the same matrices [28]. However, in that study, we did not determine whether the duration of contact with the TN-C-rich astrocytoma matrix was also associated with progressive (rather than instantaneous) impairment of the tubulogenic capacity before the endpoint at 24 h of incubation.

**Figure 7:**
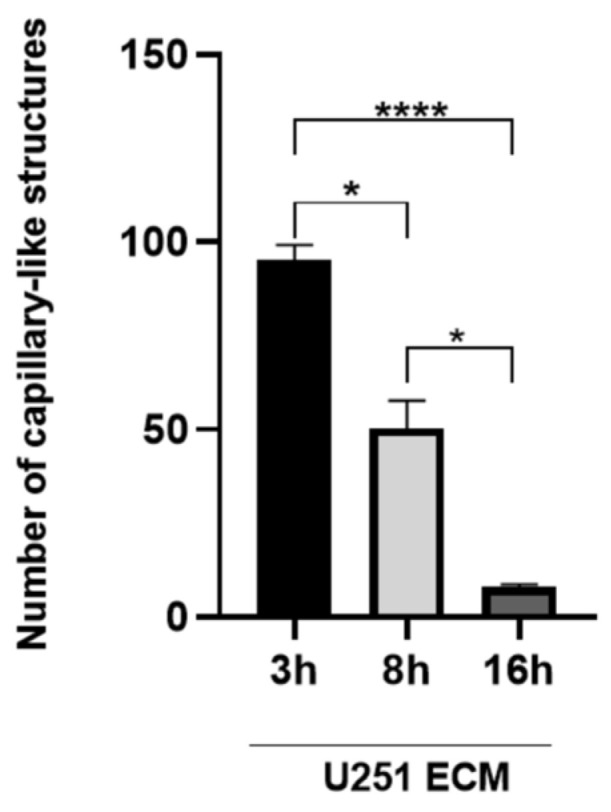
Time-course of tubulogenesis impairment of endothelial cells incubated with astrocytoma matrix. TDECs were prepared by seeding HUVECs on immobilized extracellular matrix secreted by U251-MG astrocytoma cells, as described in Section 4, and then submitted to the Matrigel™ assay in M199 supplemented with 5% FCS. After the indicated times, cells were fixed, and the total number of tube-like structures/well was counted. Data are presented as mean ± SD from three independent experiments. * *p* < 0.05, **** *p* < 0.0001.

To obtain functional evidence for gradual molecular reprogramming in response to glioma ECM, we evaluated tube formation in healthy HUVECs after different periods of preincubation (3, 6, and 16 h) on the U251-derived matrix. After 3 h of incubation with the tumor matrix (Figure 7), the endothelial cells remained competent for tube formation, consistent with the previously reported optimal adhesion at 4 h [28]. In contrast, a 47.2% inhibition of tubulogenesis was observed after 6 h, progressing to 91.6% inhibition after 16 h. Therefore, this time-dependent impairment of tubulogenic capacity supports the assumption that the TDECs transitioned gradually, rather than abruptly, to a defective angiogenic phenotype, through a process that may involve changes in protein expression.

**Figure 8:**
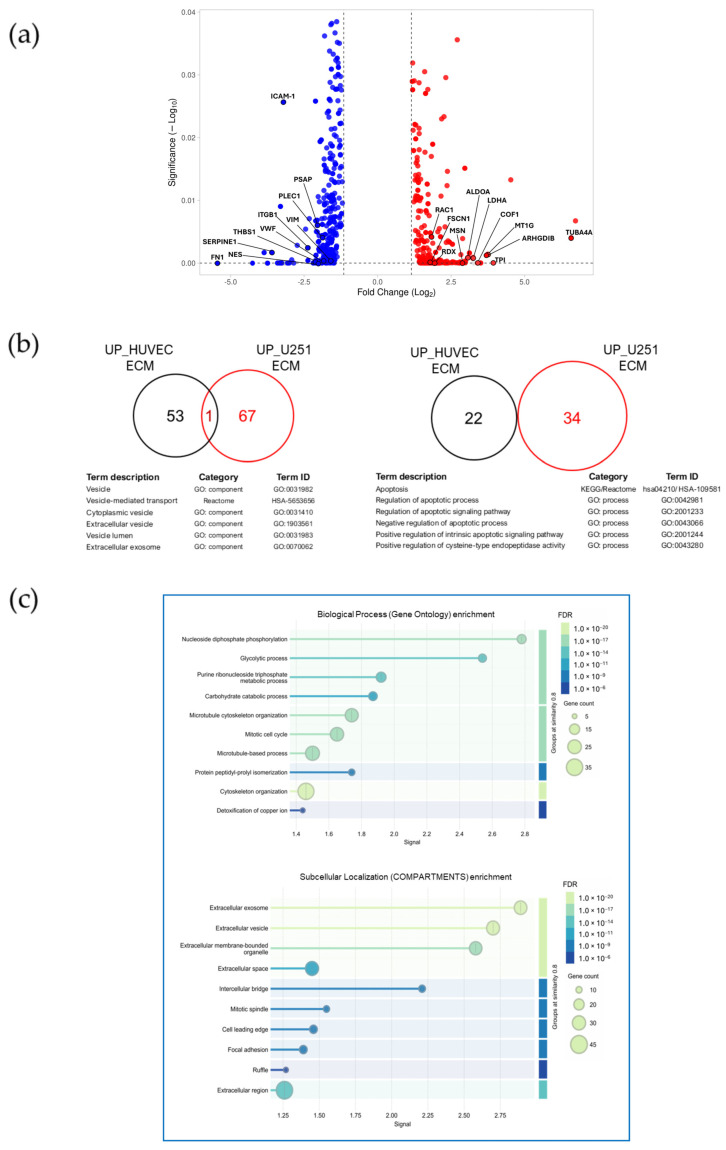
Matrix-dependent divergence of endothelial whole-cell proteomic programs. (**a**) Volcano plot showing differentially expressed proteins between upregulated (red) and downregulated (blue) proteins in tubulogenesis-defective endothelial cells (TDECs) generated after a 24-h incubation with immobilized U251-MG-derived ECM. (**b**) Venn diagrams for vesicle-related and apoptosis-related GO terms, highlighting the almost entirely non-overlapping protein sets between conditions, consistent with a matrix-imposed divergence of cellular programs, with enhanced apoptosis-related signaling, in contrast to the homeostatic profile induced by the control matrix. (**c**) Enrichment analysis summarizing the shift toward extracellular vesicle expansion, cytoskeletal remodeling, and metabolic rewiring under tumor-derived ECM.

To understand how TDECs become functionally reprogrammed to vesicle secretion and cell migration, we performed a whole-cell proteomic comparison between TDECs and control endothelial cells grown on their autologous matrices. This approach was conceived as an initial step to capture global cellular adaptations and identify signaling and regulatory pathways that could account for, or potentially drive, the secretory and migratory phenotypes observed in the preceding experiments. Whole-cell proteomic profiling of TDECs cultured on autologous or astrocytoma-derived matrices yielded a high-quality dataset suitable for downstream functional analyses. After data filtering and statistical selection (*p*-value < 0.05), a robust set of differentially expressed proteins was obtained and used for enrichment and network analyses. The resulting dataset comprised 969 identified proteins significantly modulated by the astrocytoma ECM, of which 755 were differentially abundant (fold change > 1.5; *p* < 0.05) (Figure 8a). To focus on biological interpretation, we next examined the functional categories and signaling pathways most altered in TDECs exposed to the glioma matrix (Table 1; Tables S1–S3, Supplementary Materials, for comprehensive protein lists).

Gene Ontology enrichment analyses (Table 1 and Figure 8b,c) revealed a strong bias toward categories associated with vesicle-mediated transport, extracellular vesicles, cytoskeleton organization, cell migration, apoptosis, and the glycolytic process, as well as terms related to the endoplasmic reticulum and chaperones. Among the upregulated proteins, multiple α- and β-tubulin isoforms (TUBA1A/C, TUBB3/4A/6/8) and actin-remodeling factors (PFN1, FSCN1, CFL1, WDR1) were prominent, indicating enhanced cytoskeletal dynamics compatible with vesicle trafficking and mesenchymal migratory phenotype.

We previously showed that, in addition to their impaired tubulogenic capacity, TDECs display higher proliferative activity than endothelial cells maintained for 24 h on their autologous ECM [28]. Consistent with this phenotype, subcellular localization enrichment analysis revealed proteins enriched in hallmark mitotic structures, most notably the mitotic spindle and the intercellular bridge (Figure 8c, upper panel; Figure S6, Supplementary Materials). This pattern indicates that a subset of the detected proteins is integral to the structural machinery that drives chromosome segregation and cytokinesis. In parallel, GO Biological Process enrichment (Figure 8c, lower panel) highlighted the functional dimension of this program, with dominant terms related to chromosome segregation, mitotic nuclear division, microtubule-based movement, and microtubule cytoskeleton organization. Together, these complementary enrichments demonstrate that the dataset captures both the structural components and the active molecular pathways of the G2/M cell-division machinery.

Additional upregulated groups comprised Rho-family GTPases (RHOA, RHOC, RAC1/3) and their regulators (ARHGDIA/B), together with annexins (ANXA2, ANXA5), heat-shock proteins (HSP90AA1/AB1, HSPD1), and peroxiredoxins (PRDX1/2), denoting remodeling of the vesicle-budding, redox, and chaperone systems that support secretion. (Table 1). Notably, several canonical EV marker families (HSPs, annexins, tubulins, and ARFs) were detected at elevated levels in TDECs, consistent with the enhanced EV release documented in functional assays (Table 1). In parallel, enzymes of the glycolytic pathway—including GAPDH, ALDOA, PKM, PGK1, and TPI1—were consistently increased, suggesting an energetic shift toward glycolysis that could sustain the high ATP demand of vesicle production, cytoskeletal turnover, and migration.

Across the adhesion-related GO categories, TDECs suppress an established adhesion program of endothelial cells primed with their own autologous matrix (Table 1). Core structural and anchoring components—including ITGB1 (β1-integrin), FN1 (fibronectin), THBS1, FLNA/FLNB, PLEC, MYH11, and PECAM1 were markedly reduced, together with multiple myosin light chains (MYL6, MYL9, MYL12A/B) and junctional scaffolds (DES, VIM, AHNAK).

**Figure 9:**
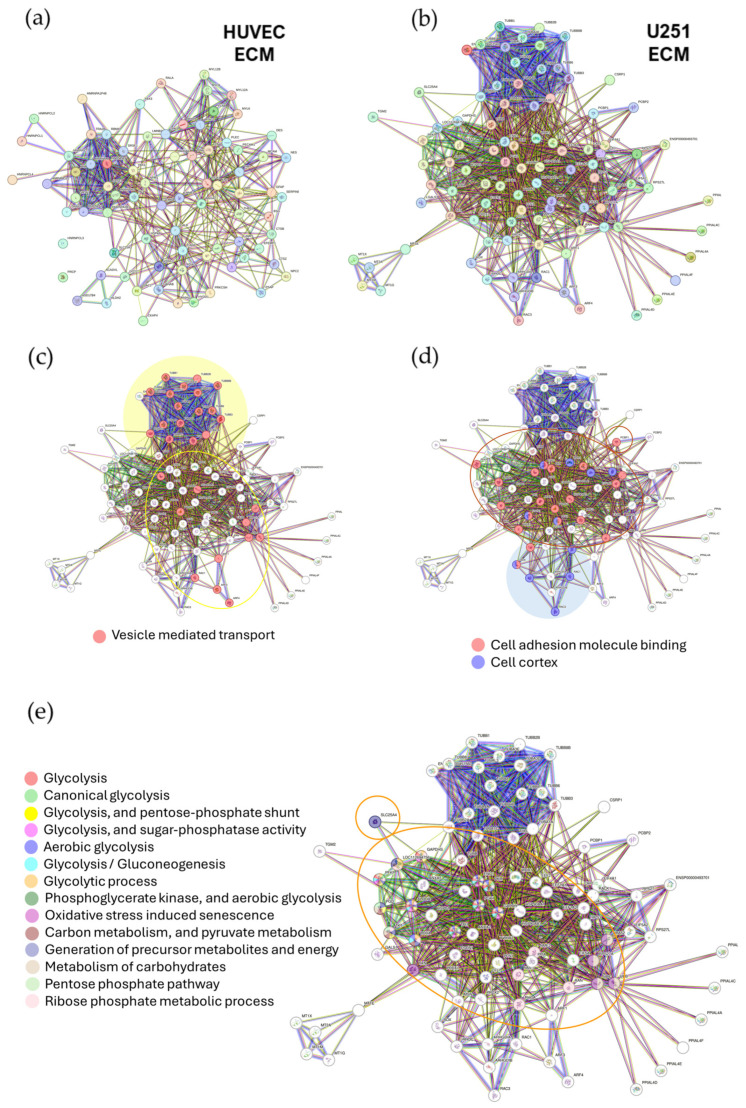
STRING network analysis of regulated proteins in tubulogenesis-defective endothelial cells (TDECs). (**a**) Protein nodes of upregulated proteins under the control condition (endothelial cells incubated with HUVEC-derived/autologous ECM) form a cohesive adhesion-cytoskeletal cluster enriched for cell–cell junctions, stress fibers, and focal adhesion nodes. (**b**) Protein nodes of upregulated proteins in HUVECs incubated with tumor-derived ECM form a radically reprogrammed network dominated by microtubule-rich modules and vesicle-trafficking hubs. In (**c**–**e**), a focus on TDECs networks is proposed. (**c**) Highlighted regions (yellow shadowing and area is outlined by an ellipse) show strong enrichment for vesicle-mediated transport; (**d**) adhesion-related clusters (cell cortex, cell–cell adhesion, cadherin/integrin binding) are depleted or displaced to peripheral positions of the TDEC STRING network. Instead, the cortical network is remodeled toward regulators of lamellipodial/podosome/leading-edge functions (RAC1/3, RHOA, WDR1, FSCN1, CAP1, CFL1, MSN), indicating a shift from a stable anchoring cortex to a protrusive, migration-oriented architecture (protein nodes encircled in dark red lines); (**e**) STRING functional overlays reveal pronounced activation of glycolysis-related pathways in TDECs, consistent with metabolic rewiring (protein nodes encircled in orange).

To contextualize the functional modules identified in the proteomic dataset, we next performed a STRING network analysis (Figure 9) comparing differentially expressed proteins (DEPs, fold change > 1.5; *p* < 0.05) in the control matrix condition (HUVEC ECM; 353 DEPs) and in the tumor-derived matrix condition (UP_U251 ECM; 402 DEPs). STRING identified 79 interaction nodes in the HUVEC ECM and 94 nodes in the U251 ECM upregulated networks, revealing a clear expansion of interconnected molecular circuitry in TDECs. These nodes confirmed that, collectively, the two conditions diverge into mutually exclusive network topologies, mirroring the functional clusters summarized in Table 1 (Figure 9; Figure S7, Supplementary Materials).

Our present results indicate that under tumor matrix cues that disorganize tubulogenesis, endothelial cells undergo a coordinated reprogramming of their cytoskeletal, metabolic, secretory, and DNA-replicative functions, integrating glycolytic activation with vesicle biogenesis, motility, and cell division. These features strongly align TDECs with the phenotypic traits of stalk- and tip-cell endothelial programs rather than with the characteristics expected of quiescent endothelial cells [34,35].

## 3. Discussion

Much of what we know about angiogenesis comes from developmental models, where vessel growth and regression follow orderly rules shaped by flow, matrix engagement, and coordinated endothelial cell (EC) behaviors [36,37,38]. In these contexts, pruning is driven mainly by reverse EC migration, with apoptosis contributing more selectively to the removal of redundant vessels [27,39]. Tumor angiogenesis, however, arises in a hypoxic, ECM-altered, and mechanically unstable microenvironment that produces disorganized vasculature not fully accounted for by developmental mechanisms [40,41]. Apoptotic cells can promote endothelial sprouting through an electrostatic, phosphatidylserine-dependent mechanism [24]. Although the study by Zhang et al. [27] provided an essential conceptual precedent, it was conducted in a non-tumor setting and was unrelated to ECM cues or the stresses characteristic of tumor tissues. In our system, we have now extended this concept into a tumor-ECM context, showing that ECM-induced endothelial apoptosis triggers a vesiculogenic, migratory phenotype in surviving cells—representing a noncanonical deployment of apoptosis compared with its classical role in developmental vascular regression.

In our current experimental set, focused on the matrix context of malignant astrocytoma, TN-C played a crucial role, as demonstrated by the partial reversion of ECM effects observed on adhesion, apoptosis, and migration when the U-251-derived ECM used to “prime” endothelial cells was depleted of TN-C (Figure 4 and Figure 6). These observations are consistent with seminal findings showing that TN-C destabilizes focal adhesions in endothelial cells [42]. TN-C is increasingly recognized as a matricellular regulator of pathological neovascularization, rather than a uniformly proangiogenic factor. It is strongly induced during development, wound repair, and cancer, where it remodels the perivascular extracellular matrix and modulates endothelial responses to angiogenic cues [43,44]. TN-C stimulates endothelial migration and can potentiate the effects of VEGF and other angiogenic growth factors [45,46,47]. At the same time, studies using domain-restricted TN-C fragments show that specific regions of the protein can exert anti-angiogenic or anti-lymphangiogenic activity [48,49,50], highlighting that TN-C functions as a context-dependent fine-tuner of vascular remodeling rather than a strictly pro- or anti-angiogenic molecule.

Beyond its direct role in regulating endothelial adhesion, proliferation, and migration, TN-C’s involvement in angiogenesis also appears to encompass more complex morphogenetic processes, primarily through its dynamic interaction with fibronectin (FN) [51,52]. While TN-C expression in stable, adult vessels is quite rare, embryonic cells lining the forming vessels consistently express both tenascin and fibronectin during vascular development [53,54,55]. This developmental frame has proven challenging to translate when examining the TN-C/FN duo’s contribution to endothelial morphogenesis in the tumor context. In our previous work, prolonged exposure to TN-C-rich matrices from high-grade astrocytomas generated endothelial cells that were more proliferative yet unable to form a branched tubular network—a phenotype fully reversed by experimentally shifting the molar balance of the two proteins in favor of FN in mixed substrata [28].

Notably, in the present study, whole-cell proteomic analysis revealed marked suppression of FN expression in tubulogenesis-defective endothelial cells (TDECs), supporting the notion that astrocytoma-derived ECM instructs endothelial cells to maintain a high TN-C-to-FN ratio, which undermines their capacity to form stable vascular tubes. Using only a purified TN-coated surface as an adhesion support, Radwanska and co-workers [56] found that HUVECs counteract the anti-adhesive activity of TN-C by increasing their expression of FN. We estimate that this divergence in results stems from the differences between the experimental models employed. Our system focuses on endothelial cells interacting with a complex glioma-derived ECM that contains multiple protein components, in addition to the prevailing TN-C. However, another study by the same group [57] reported dual effects of TN-C on angiogenesis: direct exposure to TN-C repressed endothelial adhesion, tubulogenesis, and survival. These observations corroborate our previous observations [28], although the tumor matrix-priming approach described here interrogates distinct facets of the angiogenic response.

Another original aspect of our present work was the finding that incubating healthy endothelial cells with the TN-C-rich matrix derived from U251-MG astrocytoma cells triggers a pronounced vesiculogenic activity associated with the anoikis process, thereby mitigating the tubulogenic defect of TDECs. In our initial assays, the most evident positive morphogenic outcome was an increase in the number of endothelial branch points when endothelial cells that had undergone anoikis on tumor matrix were added to the system, thereby improving the tubulogenic capacity of TDECs. Monitoring three central parameters of tubulogenesis (e.g., total tube number, tube length, and number of bifurcations) revealed that the main effect of apoptotic cells in this disorganized system was to enhance network formation, likely by stimulating the migration of putative endothelial tip cells within the TDEC population. Tip cells, the subpopulation responsible for sprouting new vascular branches at bifurcation points [29], exhibit a highly motile phenotype, driven by enhanced glycolysis [34]. Again, the whole-cell proteomic approach employed here to depict the impact of a TN-C-rich matrix on global protein expression provides strong support for TDECs as a “tip-prone” population. Moreover, these cells may coexist with “stalk-type” cells—an equally glycolytic phenotype, as evidenced by the upregulation of several essential components of the cellular replicative machinery, corroborating our previous data on the proliferative nature of TDECs [28].

Although tip and stalk cells are traditionally described as distinct endothelial phenotypes, considerable plasticity exists between them. Endothelial cells can occupy intermediate or hybrid states and transition between tip-like and stalk-like behaviors in response to VEGF gradients, mechanical cues, or Notch signaling, indicating that angiogenesis operates as a dynamic continuum rather than a set of rigid subtypes [58,59,60]. Notably, our current data suggest that the astrocytoma ECM functions as a decisive modulatory factor, profoundly influencing this phenotypic dynamism and reshaping the balance between migratory and structural (quiescent) endothelial states. In this context, future characterization of specific endothelial markers, together with the mapping of key signaling pathways implicated in tip–stalk interconversion, will be essential for determining the prevalence of transient, reversible phenotypes within the TDEC population.

Most studies that exploit the effects of EVs on endothelial differentiation focus on vesicles released directly by tumor cells [16]. A small body of evidence indicates that endothelial cells can generate EV-mediated signals that reshape endothelial phenotypes by transferring RNAs and proteins to neighboring cells [61]. For example, among the cargo molecules identified so far are delta-like 4 (Dll-4), which becomes enriched in endothelial vesicles after cells are cultured on substrates coated with immobilized Dll-4 [62], and activated STAT-5, which is selectively packaged into endothelial EVs following the stimulation of HUVECs with the soluble mediator IL-3 [63]. However, to date, no reports have described a role for the tumor matrix as the sole stimulus for endothelial vesicle secretion, which subsequently acts in an autocrine manner on the very cells that produced them, thereby enhancing endothelial morphogenesis.

The cell-level view we propose here for the modulation of endothelial cells by the astrocytoma matrix provides a mechanistic framework for the increased extracellular vesicle output and migratory behavior observed in functional assays throughout this study, thereby establishing the basis for proteomic characterization of secreted vesicles in subsequent steps of this investigation.

## 4. Materials and Methods

### 4.1. Cell Cultures

Unless otherwise stated, all culture media and supplies were from Life Technologies/Invitrogen (Thermo Scientific, São Paulo, Brazil). Human umbilical vein endothelial cells (HUVECs) were obtained by a modification of the procedure previously described [64] after treatment of umbilical veins with a 0.1% collagenase IV solution (Merck/Sigma-Aldrich, São Paulo, Brazil) and grown in 199 medium (M199, Merck/Sigma-Aldrich) supplemented with 20% fetal calf serum (FCS) and antibiotics. At least five independent primary cultures were pooled for each experiment to minimize interference from individual genetic variation. The primary endothelial cultures were generated from pools of 3 to 5 individual umbilical cords, a strategy that ensured both biological variability and donor anonymity. Cultures were used up to passage 3. The human astrocytoma cell line U251-MG (formerly distributed as U373-MG, ref.# 09063001, Merck/Sigma-Aldrich) was recently authenticated by short tandem repeat (STR) fragment analysis using the GlobalFiler™ PCR Amplification Kit (Thermo Scientific, São Paulo, Brazil) at the DNA Diagnostic Laboratory (LDD) of UERJ (Rio de Janeiro, Brazil). The STR patterns found were equivalent to those available in the database provided by the ATCC (Manassas, VA, USA) and Cellosaurus database (https://www.cellosaurus.org/ accessed on 21 October 2022). U251-MG has been classified as a malignant anaplastic astrocytoma grade III-glioblastoma in vitro that recapitulates a GBM-like tumor (grade IV) in vivo [65]. These cells were cultured in DMEM/F12 supplemented with 10% FCS, glutamine, and antibiotics.

### 4.2. Preparation of Fresh Immobilized Extracellular Matrix (ECM)

Freshly immobilized ECMs from U251-MG (tumor matrix) and endothelial cells (autologous matrix, which mediates optimal adhesion and will be used as a control condition) were obtained as previously described [28,66]. Briefly, cells were seeded onto plastic (6-, 24-, or 96-well plates), sterile glass coverslips, or 25 cm^2^ tissue culture flasks and grown to confluence (approximately 48–72 h). Monolayers were disrupted with cold extraction buffer (PBS-Ca^2+^, pH 7.4, containing 0.1% Triton X-100, 0.1 M NH_4_OH) containing a cocktail of protease inhibitors (Merck/Sigma-Aldrich). Cell debris was washed twice with cold PBS-Ca^2+^. Immobilized matrices were used as substrata for cell adhesion, viability, and long-term priming (24 h) assays with HUVECs.

### 4.3. Detection of Endothelial Cell Adhesion and Detachment-Induced Apoptosis (Anoikis)

HUVECs were seeded onto native immobilized ECMs (autologous vs. tumoral) onto 96-well plates (3 × 10^4^ cells/well, at least in triplicate) in M199/0.1% BSA for 2 or 24 h. After these periods, non-adherent endothelial cells were removed by washing with serum-free medium, and adherent cells were detected by the MTT assay, read at 595 nm on a BioRad microplate reader (Hercules, CA, USA), in parallel with a cell number standard curve (0.5–5 × 10^4^ cells/well) simultaneously seeded in M199/10% FCS. Endothelial cells that detached after 24 h from immobilized tumor ECM (i.e., cells undergoing anoikis) were collected and double-stained with annexin V-FITC conjugated (AV) (BD Biosciences, São Paulo, Brazil) and propidium iodide (PI) (Merck/Sigma-Aldrich), and cell fluorescence intensity was read using FACScalibur (Becton & Dickinson, Franklin Lakes, NJ, USA), as previously described [28].

### 4.4. Quantification of Endothelial Cells Bearing Pyknotic Nuclei

HUVECs incubated with different matrices for 24 h were fixed with 3.7% formaldehyde, permeabilized with 0.2% Triton X-100, and stained with DAPI (1 µg/mL in PBS). The number of pyknotic nuclei (PN) and intact nuclei (IN) was counted using the fluorescence digital inverted microscope EVOS-Fl (Thermo Scientific, São Paulo, Brazil) (magnification: 20×). Each condition was performed in triplicate on glass slides. Random quantification of 10 fields was performed and expressed as the ratio PN:IN.

### 4.5. In Vitro Migration Time-Lapse Video Microscopy Assays

HUVECs (2 × 10^5^ cells) were seeded onto plastic and after 24 h, serum was removed, and astrocytoma ECM-induced apoptotic HUVECs (1 × 10^5^ cells, or 50% of the number of adherent cells) were added. Image capture was performed with a digital CCD camera Hamamatsu C2400 (Artisan TG, Champaign, IL, USA) attached to a Nikon TE 300 phase-contrast optical microscope (Tokyo, Japan) at 37 °C and 5% CO_2_. For a short migration assay (approximately 10 min), a single apoptotic endothelial cell was brought into proximity (within 100 µm) of a viable endothelial monolayer using optical tweezers. A detailed description of the trapping setup and system specifications is provided elsewhere [67]. We captured one frame every 15 s. For a longer migration test (approximately 30 min), we captured one frame every 5 s.

### 4.6. Short-Term Matrigel™ Time-Lapse Video Microscopy Assay

HUVECs (2 × 10^5^ cells) were seeded onto Matrigel (growth factor-reduced, BD Biosciences, São Paulo, Brazil) with 5% FCS and incubated for 4 h before image capture. After that, glioma ECM-induced apoptotic HUVECs (1 × 10^5^ cells) were added to the culture. One frame every minute was captured.

### 4.7. Isolation of Extracellular Vesicles (EVs)

After incubating HUVECs with extracellular autologous or astrocytoma matrices for 24 h, conditioned media were collected and sequentially centrifuged at 500× *g* for 10 min, and 1000× *g* for 10 min to remove cell bodies, cellular debris, and large apoptotic bodies. The supernatant was collected and subjected to ultracentrifugation (100,000× *g*) for 4 h at 4 °C. EVs expose phosphatidylserine on their outer membrane, which allows the binding of annexin V. Therefore, EV abundance can be estimated based on annexin V-positive events. Pellets containing the microvesicle fraction were labeled with annexin V-FITC and analyzed by flow cytometry, using a CytoFLEX platform (Beckman Coulter, Indianapolis, IN, USA) as described above (Section 4.3). Calibration beads (<1 µm) were used to delimit the desired gate [68].

### 4.8. Tracking Analysis of EVs

After endothelial EV isolation, samples from each condition (derived from cells primed with autologous or tumor ECMs) were quantified by nanoparticle tracking analysis (NTA) using a Zetaview S/N 205 real-time particle analysis system (ParticleMetrix, Inning am Ammersee, Germany) according to the manufacturer’s instructions. Briefly, EV-containing samples were diluted in 1 mL PBS and injected into the sample chamber at suitable dilutions (approximately 100 samples per field) using a hypodermic syringe. Eleven fields of the sample chamber were recorded as digital video and analyzed using ZetaView 8.02.31 software at 85% sensitivity. Standardized extracellular vesicle preparations were used in subsequent experiments.

### 4.9. In Vitro Tubulogenesis Assay

HUVECs (2 × 10^5^ cells) previously incubated for 24 h with immobilized matrices secreted by the astrocytoma U251-MG (tubulogenesis-defective endothelial cells, TDECs), were seeded in triplicate wells (3 × 10^4^ cells/well) onto Matrigel™ in 96-well plates (growth factor reduced, BD Biosciences, São Paulo, Brazil, 50 µL/well) in M199 medium supplemented with 5% FCS. HUVECs seeded for the same period on their immobilized autologous matrix (competent for tube formation) were used as controls. Then, variable amounts of apoptotic HUVECs or EVs in conditioned media were added to the culture immediately. After 18 h, the 3D gels were fixed in 1.1% glutaraldehyde in PBS for 10 min, followed by three washes with PBS. Images covering the whole well area were captured using an EVOS-FL inverted microscope. The total number, length, and bifurcations of tube-like structures/wells were then counted. In some experiments, conditioned media were depleted for EVs by ultracentrifugation (100,000× *g*) for 4 h at 4 °C.

### 4.10. siRNA-Mediated Gene Silencing of Tenascin-C (TN-C)

U251-MG cells were seeded in 60-mm dishes or 6-well plates (1 × 10^4^ cells/cm^2^) in DMEM-F12 HAM supplemented with 10% FCS and 2 mM L-glutamine, without antibiotics or fungizone. Opti-MEM^®^ and Lipofectamine^®^ 2000 were obtained from Life Technologies/Invitrogen (Thermo Scientific, São Paulo, Brazil). After 24 h, cells were transfected with Lipofectamine^®^ 2000 (2 µg/mL) in Opti-MEM^®^ medium using siRNAs targeting TN-C mRNA (40 or 60 nM) or a truncated control siRNA. siRNA and Lipofectamine solutions were mixed (1:1) and incubated at room temperature for 20 min before addition to the cultures. After 24 h, the medium was replaced with complete DMEM-F12 HAM containing 10% FBS, 2 mM L-glutamine, 2.5 µg/mL fungizone, 500 U/mL penicillin, and 40 µg/mL gentamicin. Sense and antisense siRNA sequences for TN-C silencing are shown in Table 2. The stability of TN-C silencing was confirmed for up to 7 days post-transfection. The lower concentration (40 nM) was as effective as the higher concentration (60 nM), resulting in approximately 97% and 92% silencing, respectively. Therefore, we used siRNAs at 40 nM in most of the assays.

### 4.11. Western Blotting

This technique was used to confirm TN-C silencing in U251-MG cells and to verify the presence of vesicle markers in EV preparations. Briefly, U251-MG cells transfected with either the specific TN-C siRNA or a scrambled control construct were lysed in a mild buffer (50 mM Tris, pH 7.4; 150 mM NaCl; 1% Triton X-100) supplemented with a protease inhibitor cocktail (P8340, Merck/Sigma). Total protein content in the extracts was determined using the Bradford Protein Assay (BioRad Lab do Brasil, São Paulo, Brazil). Protein extracts (10 µg/lane) were resolved on a Mini-Protean TetraCell system (Bio-Rad) by SDS-PAGE using the Laemmli buffer system. Resolving gels with 6% and 10% polyacrylamide and stacking gels with 3% and 5% porosity were used to detect TN-C and β-actin, respectively, and were calibrated with full-range colored molecular weight standards (Kaleidoscope, 10–250 kDa, Bio-Rad). Proteins were transferred to PVDF membranes and probed with primary antibodies against TN-C (T2551, Merck/Sigma) and β-actin (A5441, Merck/Sigma), followed by the appropriate HRP-conjugated secondary antibodies and detection with ECL chemiluminescent reagent (Thermo Scientific). CD63 and Annexin-2 exosomal markers were separated on 12% polyacrylamide resolving gels and detected with appropriate antibodies (anti-CD63, ref. #10628D, Thermo Scientific; anti-annexin-II, ref. # sc-9061, Santa Cruz Biotech, Dallas, TX, USA).

### 4.12. Chemotaxis Assay

For cell migration (chemotaxis) analysis in vitro, tubulogenesis-defective endothelial cells (TDECs) were seeded on the upper chamber at a density of 4 × 10^4^ cells/well, in 48-well modified Boyden chambers containing a polycarbonate membrane (pore size, 8 μm; Neuroprobe, Inc., Gaithersburg, MD, USA) and allowed to migrate towards the lower chamber at 37 °C in a 5% CO_2_ humid atmosphere for 4 h. For chemotaxis, the following chemoattractant conditions were tested: (1) 10% FCS in M199, as a positive control; (2) serum-free M199, as a negative control; (3) EVs (50 EVs/μL) isolated from HUVECs from both sources (autologous ECM vs. U251-MG ECM priming), diluted in serum-free M199. Under certain conditions, TDECs were co-incubated in the upper chamber with 50 EVs/μL before migrating toward 10% FCS in M199. The cells that migrated to the lower membrane surface were fixed and stained with Wright-Giemsa, then counted under a light microscope (400×) using an Olympus BX41 (Tokyo, Japan).

### 4.13. Whole-Cell Proteomic Profiling of Tubulogenesis-Competent and Tubulogenesis-Defective Endothelial Cells

Endothelial cells were incubated for 24 h on either autologous or tumor-derived matrices. Total proteins were extracted using a lysis buffer containing 8 M urea, 1 M Tris, 4% CHAPS, and a protease inhibitor cocktail, followed by incubation at 4 °C for 30 min under gentle agitation. Interfering substances were removed by TCA/acetone precipitation [69]. Protein concentration was measured using the Qubit assay (Thermo Scientific, São Paulo, Brazil), and samples were concentrated by ultrafiltration with AMICON 3 kDa devices (Merck/Millipore, São Paulo, Brazil). After reduction and alkylation, proteins were digested with trypsin, and the resulting peptides were desalted and concentrated using OMIX C-18 mini-columns (Agilent Technologies Brasil, São Paulo). Peptides were analyzed in technical triplicates by nano-LC coupled to an LTQ Orbitrap Velos mass spectrometer (Thermo Scientific, São Paulo, Brazil) operating in data-dependent acquisition (DDA) mode, alternating between full-scan MS and MS/MS. Label-free quantification was based on extracted ion chromatograms (XIC), and differentially abundant proteins were identified using the TFold module, applying both fold-change and statistical significance thresholds. Proteins were identified using PatternLab for Proteomics (v4.0) (http://patternlabforproteomics.org, accessed on 10 August 2025) [70] against a customized database of human protein sequences downloaded from UniProt (http://www.uniprot.org, accessed on 14 April 2025). The peptide and protein false discovery rate (FDR) was set at 1% using a target–decoy strategy, and the search database included common contaminants available in PatternLab’s Search Database Generator. Searches were performed allowing tryptic and semi-tryptic peptides, up to three missed cleavages, fixed cysteine carbamidomethylation, and a precursor mass tolerance of 40 ppm. Peptide–spectrum matches (PSMs) were validated with the Search Engine Processor (SEPro) within the PatternLab environment. Identifications were grouped by charge state (2+ and 3+) and by fully tryptic or semi-tryptic status. A minimum peptide length of six amino acids was required. A Bayesian discriminator was generated for each result using ProLuCID XCorr, DeltaCN, and ZScore values. Protein identifications were accepted when supported by at least two pieces of evidence and displaying <5 ppm mass variation at the protein level. In total, 24,246 protein entries supported by at least two peptides (from a set of 9677 peptides) were identified, including an estimated ~25% redundancy attributable to peptide sharing among isoform- or paralog-related entries. Differentially expressed proteins were analyzed with STRING v.12.0 (https://string-db.org, accessed on 17 September 2025) using default parameters, and functional enrichments were evaluated for their relevance to endothelial remodeling. A volcano plot was generated using VolcaNoseR (https://huygens.science.uva.nl/VolcaNoseR/, accessed on 17 September 2025) to visualize statistically significant protein expression changes (fold change > 1.5; *p* < 0.05).

### 4.14. Statistical Analysis

Data are presented as the mean ± standard deviation (SD) from at least three independent experiments, as indicated in the figure’s captions. Normality was assessed for all datasets using the Shapiro–Wilk test. Comparisons between two groups used an unpaired two-tailed *t*-test (or Mann–Whitney test for non-parametric data). Comparisons among more than two groups used either a one-way ANOVA or the Kruskal–Wallis test for nonparametric data, followed by appropriate post hoc tests (Tukey’s or Dunn’s tests). Statistical analyses were performed in GraphPad Prism 5.01.

## Figures and Tables

**Table 1:** Core Functional Signatures Defining the Proteome of Tubulogenesis-Defective Endothelial Cells (TDECs).

Enriched functional Categories:	Protein Symbols (Representative Set)	UniProt Accession/Protein Names (Selected Top Modulated Core Proteins)	Fold Change	*p*-Value
1. Migration & cytoskeletal remodeling (lamellipodia/filopodia/podosome/leading edge dynamics)	PFN1, RAC1, RAC3, RHOC, RHOA, ARHGDIA/B, FSCN1, CFL1, WDR1, CAP1, MSN, RDX, TUBB1/2A/2B/3/4A/4B/6/8/8B, TUBA1A/1B/1C/3D/3E/4A/8, NME1/2, RPS3, EEF1A1, YWHAE/Z	C9JDS9 (Tubulin alpha-4A chain)	6.58	0.00404
		F5H2R5 (Rho GDP-dissociation inhibitor 2)	3.70	0.00128
		V9GZ54 (Moesin)	2.91	0.00001
		H0YB80 (14-3-3 protein zeta/delta)	2.54	0.00301
		Q16658 (Fascin)	1.85	0.00012
		P07737 (Profilin-1)	1.71	0.00130
2. Vesicle biogenesis, trafficking & secretion	ARF1, ARF3, ARF4, HSP90AA1, HSP90AB1, ANXA2, ANXA5, TUBB isoforms, TUBA isoforms, CAP1, MSN, RAN, RDX, TXN, PRDX1/2, PPIA, PCBP2	F8WE65 (Peptidyl-prolyl cis-trans isomerase)	3.51	0.00002
		B4DNG6 (Annexin)	2.32	0.00005
		P35241 (Radixin)	2.06	0.00001
		Q06830 (Peroxiredoxin-1)	1.87	0.00003
		P84077 (ADP-ribosylation factor-1)	1.60	0.00340
3. Glucose metabolism & energy supply	ENO1, ALDOA, PKM, LDHA, PFKP, GAPDH, TPI1, PGK1, PGD, TKT, SLC25A4	A0A0S2Z359 (Solute carrier family 25 member 4)	6.73	0.00674
		Q2QD09 (Triose-phosphate isomerase)	3.94	0.00002
		K7EM49 (6-phosphogluconate dehydrogenase)	3.30	0.00001
		F5GXH2 (L-lactate dehydrogenase)	3.25	0.00092
		P14618 (Pyruvate kinase M)	3.11	0.00001
4. Anoikis priming (reversible stress-adaptive response and cytoskeletal–oxidative interplay)	ANXA5, RPS3, EIF5A, PPIA, PRDX2, RACK1, ARHGDIA, ENO1, SLC25A4, YWHAE/Z, HSP90AA1, HSP90AB1, HSPD1, RHOA, RHOC, NME1, NME2	C9J0S9 (60 kDa heat shock protein, mitochondrial)	2.71	0.03560
		E7ERL0 (Nucleoside diphosphate kinase A)	2.38	0.00002
		I3L397 (Eukaryotic translation initiation factor 5A)	2.14	0.00420
		P08758 (Annexin A5)	2.07	0.00002
		E9PQX2 (40S Ribosomal protein S3)	1.59	0.00595
**Under-represented** **functional categories:**				
Cell adhesion & cytoskeletal anchoring machinery	AHNAK, ANXA2, BSG, CALR, CANX, CD59, CLU, DES, FN1, FLNA, FLNB, GFAP, HSPA9, ITGB1, LAMP1, LMNA, LMNB1, LMNB2, MCAM, MYH11, MYH9, MYL12A, MYL12B, MYL6, MYL9, P4HB, PDIA3, PECAM1, PLEC, RALA, THBS1, TPM3, TPM4, VDAC1, VIM, VWF, YBX3	Q9H382 (Fibronectin-1)	−5.44	0.00001
		C9JJP8 (Integrin β1)	−3.30	0.00090
		P07996 (Thrombospondin-1)	−2.11	0.00042
		D6R904 (Tropomyosin alpha-3 chain)	−2.02	0.01059
		I3L192 (Basigin)	−1.98	0.00019
		P04275 (von Willebrand factor)	−1.80	0.00062
		A0A075B727 (Platelet endothelial cell adhesion molecule)	−1.70	0.00376
		E9PBF6 (Lamin B1)	−1.61	0.00652
		W8QEH3 (Lamin A/C)	−1.55	0.00418

Key for protein names (except for those highlighted in the UniProt accession column, above): AHNAK—AHNAK nucleoprotein; ALDOA—Fructose-bisphosphate aldolase A; ANXA2—Annexin 2; ARHGDIA/B—Rho GDP-dissociation inhibitor 1/2; CALR—Calreticulin; CANX—Calnexin; CAP1—Adenylyl cyclase–associated protein 1; CD59—CD59 glycoprotein (protectin); CFL1—Cofilin-1; CLU—Clusterin (Apolipoprotein J); DES—Desmin; EEF1A1—Elongation factor 1-alpha 1; ENO1—Alpha-enolase; FLNA/B—Filamin-A/B; GAPDH—Glyceraldehyde-3-phosphate dehydrogenase; GFAP—Glial fibrillary acidic protein; HSPA9—Stress-70 protein, mitochondrial (GRP75/mortalin); HSP90AA1—Heat shock protein HSP 90-alpha; HSP90AB1—Heat shock protein HSP 90-beta; LAMP1—Lysosome-associated membrane glycoprotein 1; MCAM—Melanoma cell adhesion molecule (CD146); MYH11—Myosin-11; MYH9—Myosin-9; MYL6—Myosin light chain 6; MYL9—Myosin regulatory light chain 9; MYL12A—Myosin regulatory light chain 12A; MYL12B—Myosin regulatory light chain 12B; NME2—Nucleoside diphosphate kinase B; P4HB—Protein disulfide-isomerase; PDIA3—Protein disulfide-isomerase A3; PECAM1—Platelet endothelial cell adhesion molecule (CD31); PFKP—6-phosphofructokinase, platelet isoform; PGK1—Phosphoglycerate kinase 1; PKM—Pyruvate kinase PKM; PLEC—Plectin; PPIA—Peptidyl-prolyl cis-trans isomerase A; PRDX2—Peroxiredoxin-2; RACK1—Receptor for activated C kinase 1 (GNB2L1); RAC1—Ras-related C3 botulinum toxin substrate 1; RAC3—Ras-related C3 botulinum toxin substrate 3; RALA—Ras-related protein Ral-A; RHOA—Transforming protein RhoA; RHOC—Rho-related GTP-binding protein RhoC; TKT—Transketolase; TPI1—Triosephosphate isomerase; TPM3—Tropomyosin alpha-3 chain; TPM4—Tropomyosin alpha-4 chain; TUBA—Tubulin alpha chains; TUBB—Tubulin beta chains; VDAC1—Voltage-dependent anion-selective channel protein 1; VIM—Vimentin; WDR1—WD repeat-containing protein 1; YBX3—Y-box-binding protein 3.

**Table 2:** Sequence and Design of siRNAs Used for TN-C Gene Silencing.

siRNA ID	Sense Sequence (5′–3′)	Antisense Sequence (5′–3′)	Concentrations (nM)
TN-C siRNA	5′-CAGCCAGUGGUGUUUAACCACGUUU-3′	5′-AAACGUGGUUAAACACCACUGGCUG-3′	40, 60
Control siRNA	5′-CAGGUGAUGUGAAUUCACCGCCUUU-3′	5′-AAAGGCGGUGAAUUCACAUCACCUG-3′	40, 60

## Data Availability

The original contributions presented in this study are included in the article/Supplementary Materials. Further inquiries can be directed to the corresponding author. The raw data supporting the conclusions of this article will be made available by the authors on request.

## Supplementary Materials

The following supporting information can be downloaded at: https://www.mdpi.com/article/10.3390/ijms27062574/s1.

## Abbreviations

The following abbreviations are used in this manuscript:

DEP	Differentially expressed proteins
ECM	Extracellular matrix
EV	Extracellular vesicle
FN	Fibronectin
GO	Gene Ontology
HUVECs	Human umbilical vein endothelial cells
NTA	Nanoparticle tracking analysis
TDECs	Tubulogenesis-defective endothelial cells
TN-C	Tenascin-C

## References

[1] Liu Z., Chen H., Zheng L., Sun L., Shi L. (2023). Angiogenic signaling pathways and anti-angiogenic therapy for cancer. Signal Transduct. Target. Ther..

[2] Rhun E., Preusser M., Roth P., Reardon D.A., Bent M.V.D., Wen P., Reifenberger G., Weller M. (2019). Molecular targeted therapy of glioblastoma. Cancer Treat. Rev..

[3] Weller M., Wick W., Aldape K., Brada M., Berger M., Pfister S.M., Nishikawa R., Rosenthal M., Wen P.Y., Stupp R. (2015). Glioma. Nat. Rev. Dis. Primers.

[4] Yang K., Wu Z., Zhang H., Zhang N., Wu W., Wang Z., Dai Z., Zhang X., Zhang L., Peng Y. (2022). Glioma targeted therapy: Insight into future of molecular approaches. Mol. Cancer.

[5] Broekman M.L., Maas S.L.N., Abels E.R., Mempel T.R., Krichevsky A.M., Breakefield X.O. (2018). Multidimensional communication in the microenvirons of glioblastoma. Nat. Rev. Neurol..

[6] Hadi L.A., Anelli V., Guarnaccia L., Navone S., Beretta M., Moccia F., Tringali C., Urechie V., Campanella R., Marfia G. (2018). A bidirectional crosstalk between glioblastoma and brain endothelial cells potentiates the angiogenic and proliferative signaling of sphingosine-1-phosphate in the glioblastoma microenvironment. BBA—Mol. Cell Biol. Lipids.

[7] Kalluri R., McAndrews K.M. (2023). The role of extracellular vesicles in cancer. Cell.

[8] Kalluri R., LeBleu V.S. (2020). The biology, function, and biomedical applications of exosomes. Science.

[9] Hauser P., Wang S., Didenko V.V. (2017). Apoptotic bodies: Selective detection in extracellular vesicles: Selective detection in extracellular vesicles. Methods Mol. Biol..

[10] Van Niel G., D’angelo G., Raposo G. (2018). Shedding light on the cell biology of extracellular vesicles. Nat. Rev. Mol. Cell Biol..

[11] Nogués L., Benito-Martin A., Hergueta-Redondo M., Peinado H. (2018). The influence of tumour-derived extracellular vesicles on local and distal metastatic dissemination. Mol. Asp. Med..

[12] Abels E.R., Breakefield X.O. (2016). Introduction to extracellular vesicles: Biogenesis, RNA cargo selection, content, release, and uptake. Cell Mol. Neurobiol..

[13] Giusti I., Delle Monache S., Di Francesco M., Sanità P., D’Ascenzo S., Gravina G.L., Festuccia C., Dolo V. (2016). From glioblastoma to endothelial cells through extracellular vesicles: Messages for angiogenesis. Tumour Biol..

[14] Ko S.Y., Lee W., Kenny H.A., Dang L.H., Ellis L.M., Jonasch E., Lengyel E., Naora H. (2019). Cancer-derived small extracellular vesicles promote angiogenesis by heparin-bound, bevacizumab-insensitive VEGF, independent of vesicle uptake. Commun. Biol..

[15] Kuriyama N., Yoshioka Y., Kikuchi S., Azuma N., Ochiya T. (2020). Extracellular Vesicles Are Key Regulators of tumor neovasculature. Front. Cell Dev. Biol..

[16] Kugeratski F.G., Santi A., Zanivan S. (2022). Extracellular vesicles as central regulators of blood vessel function in cancer. Sci. Signal..

[17] Mathiesen A., Hamilton T., Carter N., Brown M., McPheat W., Dobrian A. (2021). Endothelial extracellular vesicles: From keepers of health to messengers of disease. Int. J. Mol. Sci..

[18] Banizs A.B., Huang T., Nakamoto R.K., Shi W., He J. (2018). Endocytosis pathways of endothelial cell-derived exosomes. Mol. Pharm..

[19] Dignat-George F., Boulanger C.M. (2011). The many faces of endothelial microparticles. Arterioscler. Thromb. Vasc. Biol..

[20] Gregory C.D., Ford C.A., Voss J.J.L.P., Gregory C. (2016). Microenvironmental Effects of Cell Death in Malignant Disease. Advances in Experimental Medicine and Biology.

[21] Gregory C.D., Rimmer M.P. (2023). Extracellular vesicles arising from apoptosis: Forms, functions, applications. J. Pathol..

[22] Chen Z., Han F., Du Y., Shi H., Zhou W. (2023). Hypoxic microenvironment in cancer: Molecular mechanisms and therapeutic interventions. Signal Transduct. Target. Ther..

[23] Winkler J., Abisoye-Ogunniyan A., Metcalf K.J., Werb Z. (2020). Concepts of extracellular matrix remodelling in tumour progression and metastasis. Nat. Commun..

[24] Weihua Z., Tsan R., Schroit A.J., Fidler I.J. (2005). Apoptotic cells initiate endothelial cell sprouting via electrostatic signaling. Cancer Res..

[25] Korn C., Augustin H.G. (2015). Mechanisms of vessel pruning and regression. Dev. Cell.

[26] Watson E.C., Koenig M.N., Grant Z.L., Whitehead L., Trounson E., Dewson G., Coultas L. (2016). Apoptosis regulates endothelial cell number and capillary vessel diameter but not vessel regression during retinal angiogenesis. Development.

[27] Zhang Y., Xu B., Chen Q., Yan Y., Du J., Du X. (2018). Apoptosis of endothelial cells contributes to brain vessel pruning of zebrafish during development. Front. Mol. Neurosci..

[28] Alves T.R., Fonseca A.C.C., Nunes S.S., Silva A.O., Dubois L.G.F., Faria J., Kahn S.A., Viana N.B., Marcondes J., Legrand C. (2011). Tenascin-C in the extracellular matrix promotes the selection of highly proliferative and tubulogenesis-defective endothelial cells. Exp. Cell Res..

[29] Mada J., Tokihiro T. (2022). Pattern formation of vascular network in a mathematical model of angiogenesis. Jpn. J. Ind. Appl. Math..

[30] Ciardiello C., Cavallini L., Spinelli C., Yang J., Reis-Sobreiro M., Candia P., Minciacchi V., Vizio D. (2016). Focus on extracellular vesicles: New frontiers of cell-to-cell communication in cancer. Int. J. Mol. Sci..

[31] Camus S.M., Gausserès B., Bonnin P., Loufrani L., Grimaud L., Charue D., De Moraes J.A., Renard J.M., Tedgui A., Boulanger C.M. (2012). Erythrocyte microparticles can induce kidney vaso-occlusions in a murine model of sickle cell disease. Blood.

[32] Camus S.M., De Moraes J.A., Bonnin P., Abbyad P., Le Jeune S., Lionnet F., Loufrani L., Grimaud L., Lambry J.C., Charue D. (2015). Circulating cell membrane microparticles transfer heme to endothelial cells and trigger vasoocclusions in sickle cell disease. Blood.

[33] Liu Y., Wang C. (2023). A review of the regulatory mechanisms of extracellular vesicles-mediated intercellular communication. Cell Commun. Signal..

[34] Eelen G., Treps L., Li X., Carmeliet P. (2020). Basic and Therapeutic Aspects of Angiogenesis Updated. Circ. Res..

[35] Ricard N., Bailly S., Guignabert C., Simons M. (2021). The quiescent endothelium: Signalling pathways regulating organ-specific endothelial normalcy. Nat. Rev. Cardiol..

[36] Franco C.A., Jones M.L., Bernabeu M.O., Geudens I., Mathivet T., Rosa A., Lopes F.M., Lima A.P., Ragab A., Collins R.T. (2015). Dynamic endothelial cell rearrangements drive developmental vessel regression. PLoS Biol..

[37] Ricard N., Simons M. (2015). When it is better to regress: Dynamics of vascular pruning. PLoS Biol..

[38] Lenard A., Daetwyler S., Betz C., Ellertsdottir E., Belting H.G., Huisken J., Affolter M. (2015). Endothelial cell self-fusion during vascular pruning. PLoS Biol..

[39] Tisch N., Almodóvar C.R. (2021). Contribution of cell death signaling to blood vessel formation. Cell. Mol. Life Sci..

[40] Jin Y., Jakobsson L. (2012). The dynamics of developmental and tumor angiogenesis—A comparison. Cancers.

[41] Martin J.D., Seano G., Jain R.K. (2019). Normalizing function of tumor vessels: Progress, opportunities, and challenges. Annu. Rev. Physiol..

[42] Chung C.Y., Murphy-Ullrich J.E., Erickson H.P. (1996). Mitogenesis, cell migration, and loss of focal adhesions induced by tenascin-C interacting with its cell surface receptor, annexin II. Mol. Biol. Cell.

[43] Orend G., Chiquet-Ehrismann R. (2006). Tenascin-C induced signaling in cancer. Cancer Lett..

[44] Midwood K.S., Orend G. (2009). The role of tenascin-C in tissue injury and tumorigenesis. J. Cell Commun. Signal..

[45] Schenk S., Chiquet-Ehrismann R., Battegay E.J. (1999). The fibrinogen globe of tenascin-C promotes basic fibroblast growth factor-induced endothelial cell elongation. Mol. Biol. Cell.

[46] Ballard V.L.T., Sharma A., Duignan I., Holm J.M., Chin A., Choi R., Hajjar K.A., Wong S., Edelberg J.M. (2006). Vascular tenascin-C regulates cardiac endothelial phenotype and neovascularization. FASEB J..

[47] Tanaka K., Hiraiwa N., Hashimoto H., Yamazaki Y., Kusakabe M. (2004). Tenascin-C regulates angiogenesis in tumor through the regulation of vascular endothelial growth factor expression. Int. J. Cancer.

[48] Saito Y., Imazeki H., Miura S., Yoshimura T., Okutsu H., Harada Y., Ohwaki T., Nagao O., Kamiya S., Hayashi R. (2007). A peptide derived from tenascin-C induces beta1 integrin activation through syndecan-4. J. Biol. Chem..

[49] Saito Y., Shiota Y., Nishisaka M., Owaki T., Shimamura M., Fukai F. (2008). Inhibition of angiogenesis by a tenascin-C peptide which is capable of activating beta1-integrins. Biol. Pharm. Bull..

[50] Katoh D., Senga Y., Mizutani K., Maruyama K., Yamakawa D., Yamamuro T., Hiroe M., Yamanaka K., Sudo A., Katayama N. (2025). Negative regulation of lymphangiogenesis by tenascin-C delays the resolution of inflammation. iScience.

[51] Van Obberghen-Schilling E., Tucker R.P., Saupe F., Gasser I., Cseh B., Orend G. (2011). Fibronectin and tenascin-C: Accomplices in vascular morphogenesis during development and tumor growth. Int. J. Dev. Biol..

[52] Morandi V., Fernandes L.R., Silva-de-Barros A.O., Papadimitriou E., Mikelis C.M. (2022). Functional Interplay Between Fibronectin and Matricellular Proteins in the Control of Endothelial Tubulogenesis. Matrix Pathobiology and Angiogenesism.

[53] Spence S.G., Poole T.J. (1994). Developing blood vessels and associated extracellular matrix as substrates for neural crest migration in Japanese quail, *Coturnix coturnix japonica*. Int. J. Dev. Biol..

[54] Astrof S., Hynes R.O. (2009). Fibronectins in vascular morphogenesis. Angiogenesis.

[55] Imanaka-Yoshida K., Nakanishi T., Markwald R., Baldwin H., Keller B., Srivastava D., Yamagishi H. (2016). Extracellular Matrix Remodeling in Vascular Development and Disease. Etiology and Morphogenesis of Congenital Heart Disease.

[56] Radwanska A., Grall D., Schaub S., Beghelli-de la Forest D.S., Ciais D., Rekima S., Rupp T., Sudaka A., Orend G., Van Obberghen-Schilling E. (2017). Counterbalancing anti-adhesive effects of tenascin-C through fibronectin expression in endothelial cells. Sci. Rep..

[57] Rupp T., Langlois B., Koczorowska M.M., Radwanska A., Sun Z., Hussenet T., Lefebvre O., Murdamoothoo D., Arnold C., Klein A. (2016). Tenascin-C orchestrates glioblastoma angiogenesis by modulation of pro- and anti-angiogenic signaling. Cell Rep..

[58] Goveia J., Rohlenova K., Taverna F., Treps L., Conradi L.C., Pircher A., Geldhof V., de Rooij L.P., Kalucka J., Sokol L. (2020). An integrated gene expression landscape profiling approach to identify lung tumor endothelial cell heterogeneity and angiogenic candidates. Cancer Cell.

[59] Venkatraman L., Regan E.R., Bentley K. (2016). Time to Decide? Dynamical Analysis Predicts Partial Tip/Stalk Patterning States Arise during Angiogenesis. PLoS ONE.

[60] Bentley K., Philippides A., Regan E.R. (2014). Do endothelial cells dream of eclectic shape?. Dev. Cell.

[61] Todorova D., Simoncini S., Lacroix R., Sabatier F., Dignat-George F. (2017). Extracellular vesicles in angiogenesis. Circ. Res..

[62] Sheldon H., Heikamp E., Turley H., Dragovic R., Thomas P., Oon C.E., Leek R., Edelmann M., Kessler B., Sainson R.C. (2010). New mechanism for Notch signaling to endothelium at a distance by delta-like 4 incorporation into exosomes. Blood.

[63] Lombardo G., Dentelli P., Togliatto G., Rosso A., Gili M., Gallo S., Deregibus M.C., Camussi G., Brizzi M.F. (2016). Activated Stat5 trafficking via endothelial cell-derived extracellular vesicles controls IL-3 pro-angiogenic paracrine action. Sci. Rep..

[64] Jaffe E.A., Nachman R.L., Becker C.G., Minick C.R. (1973). Culture of human endothelial cells derived from umbilical veins. Identification by morphologic and immunologic criteria. J. Clin. Investig..

[65] Jacobs V.L., Valdes P.A., Hickey W.F., De Leo J.A. (2011). Current review of in vivo GBM rodent models: Emphasis on the CNS-1 tumour model. ASN Neuro.

[66] Morandi V., Cherradi S.E., Lambert S., Fauvel-Lafève F., Legrand Y.J., Legrand C. (1994). Proinflammatory cytokines (interleukin-1 beta and tumor necrosis factor-alpha) downregulate synthesis and secretion of thrombospondin by human endothelial cells. J. Cell. Physiol..

[67] Pompeu P., Lourenço P.S., Ether D.S., Soares J., Farias J., Maciel G., Viana N.B., Nussenzveig H.M., Pontes B. (2021). Protocol to measure the membrane tension and bending modulus of cells using optical tweezers and scanning electron microscopy. STAR Protoc..

[68] Rautou P.E., Vion A.C., Amabile N., Chironi G., Simon A., Tedgui A., Boulanger C.M. (2011). Microparticles, Vascular Function, and Atherothrombosis. Circ. Res..

[69] Yuan X., Russell T., Wood G., Desiderio D.M. (2002). Analysis of the human lumbar cerebrospinal fluid proteome. Electrophoresis.

[70] Carvalho P.C., Lima D.B., Leprevost F.V., Santos M.D., Fischer J.S., Aquino P.F., Moresco J.J., Yates J.R., Barbosa V.C. (2016). Integrated analysis of shotgun proteomic data with PatternLab for proteomics 4.0. Nat. Protoc..

