# Supplementary material for: Proangiogenic Properties of Extracellular Vesicles Secreted by Endothelial Cells Reversibly Primed for Anoikis: A Possible Autocrine Mechanism Induced by Astrocytoma Extracellular Matrix
Source: Int J Mol Sci. 2026 Mar 11;27(6):2574. doi: 10.3390/ijms27062574 (PMC13026904; doi:10.3390/ijms27062574)
Supplement: Supplementary file 1 [file ijms-27-02574-s001.zip › Description of supplementary tables key terms and information.pdf]

Supplementary tables content - Key terms and information

Manuscript title:
"Proangiogenic properties of extracellular vesicles secreted by endothelial cells reversibly primed for anoikis: a possible autocrine mechanism induced by astrocytoma extracellular matrix"

By Silva-de-Barros *et al.*

Table S1.
DEP broad list_U251 ECM_*vs*_HUVEC ECM
Content: a table containing four worksheets. The fourth sheet represents the differentially expressed proteins (DEP) selected using the TFold module in PatternLab for Proteomics with the following thresholds: an F-stringency of 0.02, which imposes a stringent combined requirement for fold change and statistical consistency; a Q-value cutoff of 0.05 to control the false discovery rate associated with multiple testing; and a protein-level FDR of 0.05 to ensure high-confidence identifications. The following notations can be read in this worksheet: UP_PMU: upregulated proteins in the U251 ECM condition; UP_PMHV: upregulated proteins in the HUVEC ECM condition.

Table S2.
STRING analysis_UP_U251 ECM
Content: list of upregulated proteins used for analyzing enrichment categories in GO, Reactome, KEGG and InterPro databases by the open tool STRING v12.0 (https://string-db.org/) using default parameters, in the experimental condition U251 ECM (tumor matrix).

Table S3.
STRING analysis_UP_HUVEC ECM
Content: list of upregulated proteins used for analyzing enrichment categories in GO, Reactome, KEGG and InterPro databases by the open tool STRING v12.0 (https://string-db.org/) using default parameters, in the experimental condition HUVEC ECM (tumor matrix).
