# Supplementary material for: Proangiogenic Properties of Extracellular Vesicles Secreted by Endothelial Cells Reversibly Primed for Anoikis: A Possible Autocrine Mechanism Induced by Astrocytoma Extracellular Matrix
Source: Int J Mol Sci. 2026 Mar 11;27(6):2574. doi: 10.3390/ijms27062574 (PMC13026904; doi:10.3390/ijms27062574)
Supplement: Supplementary file 1 [file ijms-27-02574-s001.zip › Supplementary Material file - Version 2 - Jan 2026.pdf]

Supplementary Figures

Manuscript:
"Proangiogenic properties of extracellular vesicles secreted by endothelial cells reversibly primed for *anoikis*: a possible autocrine mechanism induced by astrocytoma extracellular matrix".

By Silva-de-Barros et al.

Content:

Figure S1 – Untagged Figure 1.

Figure S2 - Morphological analysis of tubulogenesis.

Figure S3 - Morphological aspect of picnotic nuclei.

Figure S4 - Analysis of TN-C silencing in U251-MG cells by western blotting.

Figure S5 - Size distribution of EVs assessed by NTA and marker validation by Western blotting

Figure S6 - Enrichment analysis of GO terms related to cellular components and protein domains and features (InterPro) in the proteome of TDECs.

Figure S7 - STRING network analysis of upregulated proteins in endothelial cells primed with autologous ECM (HUVEC ECM condition): a focus on cell adhesion-related nodes.

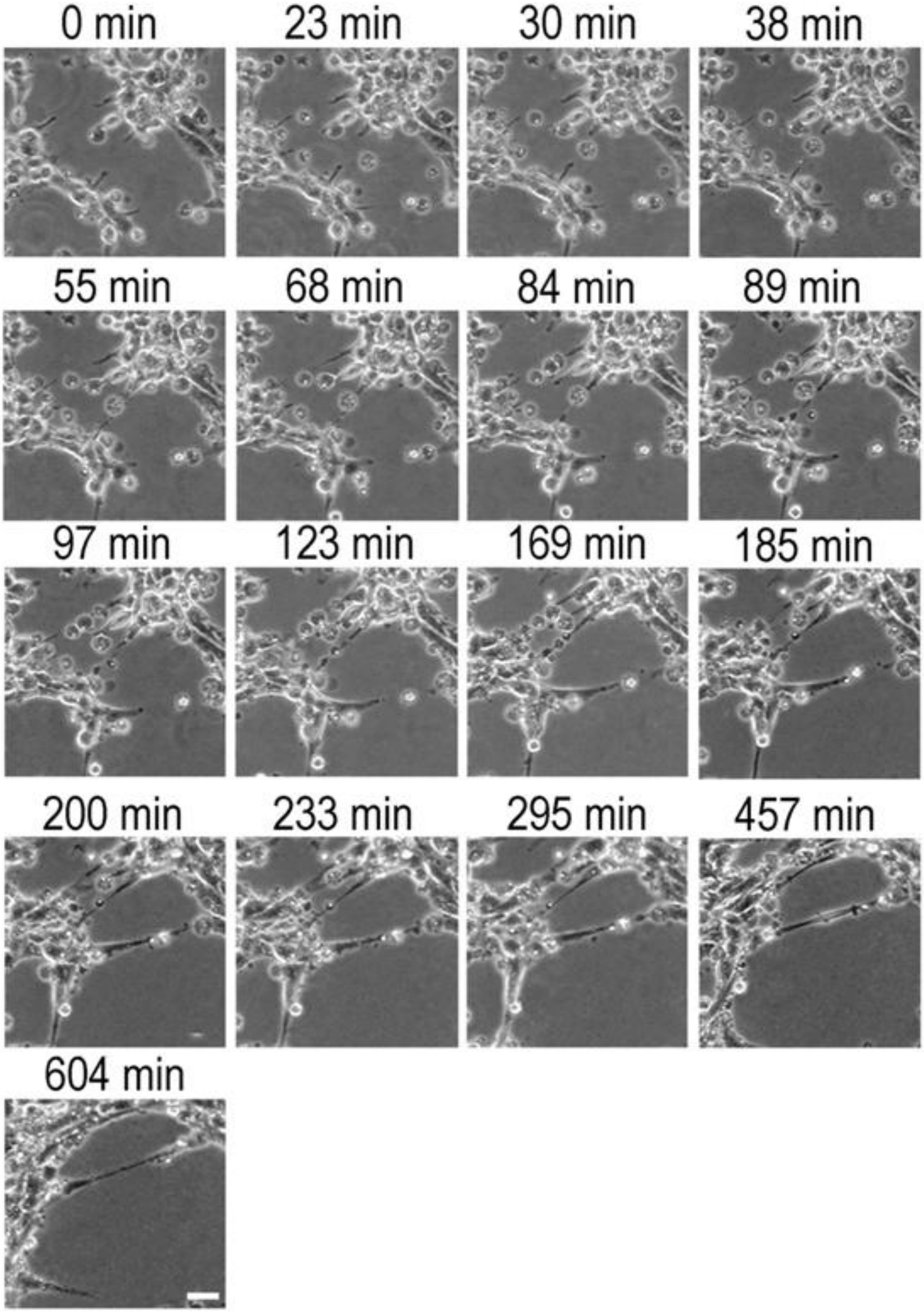

Figure S1 – Untagged Figure 1.

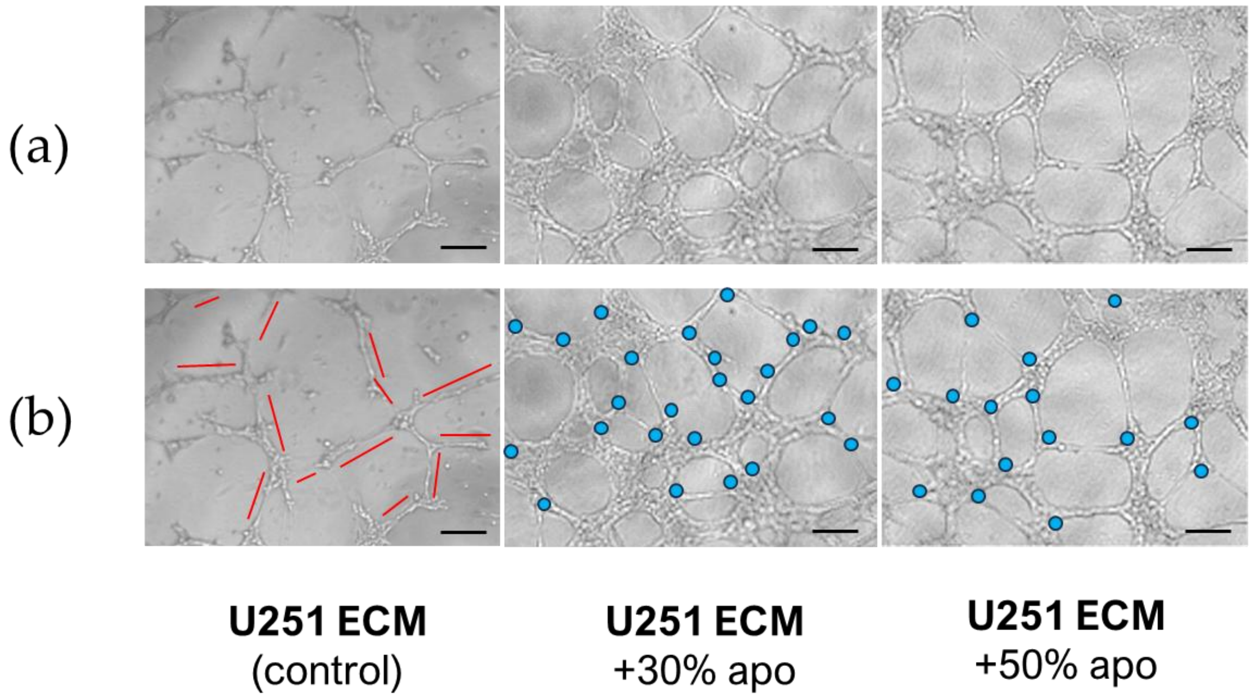

Figure S2 - Morphological analysis of tubulogenesis. TDECs were obtained by incubating primary endothelial cells on immobilized U251-MG ECM for 24 hours. TDECs were plated on Matrigel™ plugs for 16 hours, in the absence or presence of unfractionated conditioned media collected from TDECs, containing endothelial apoptotic cells, as described in the Methods section. (a) Untagged images; (b) Examples of quantification of sprout number/extension (red lines) and endothelial bifurcations (blue circles) in three different conditions.

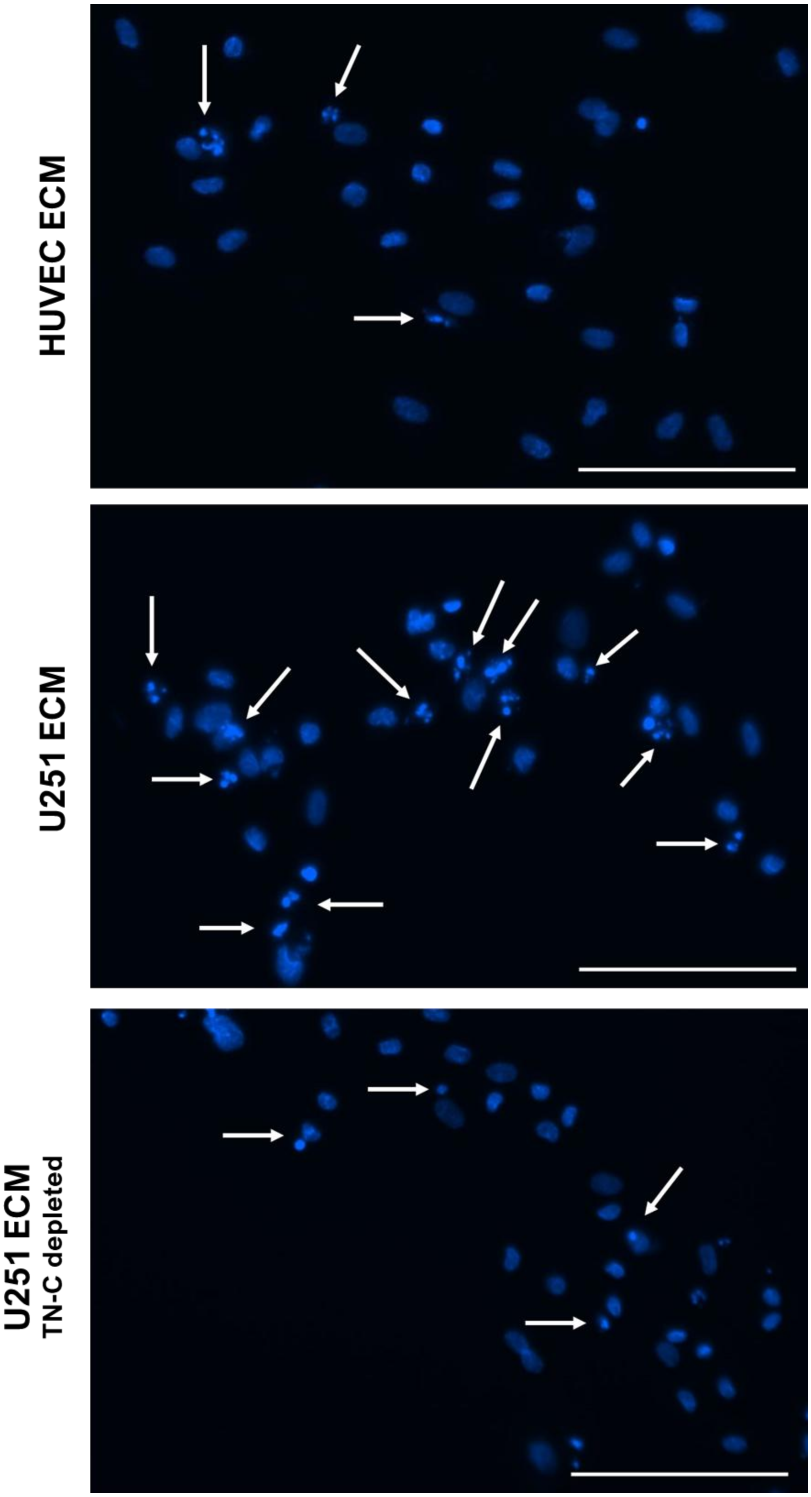

Figure S3 – Morphological analysis of pyknotic nuclei. TDECs were obtained by incubating primary endothelial cells on immobilized U251 ECM. Endothelial cells incubated with their own ECM served as controls (HUVEC ECM). After 24 hours, adhering cells were stained with DAPI and analyzed for the presence of pyknotic nuclei in the indicated conditions.

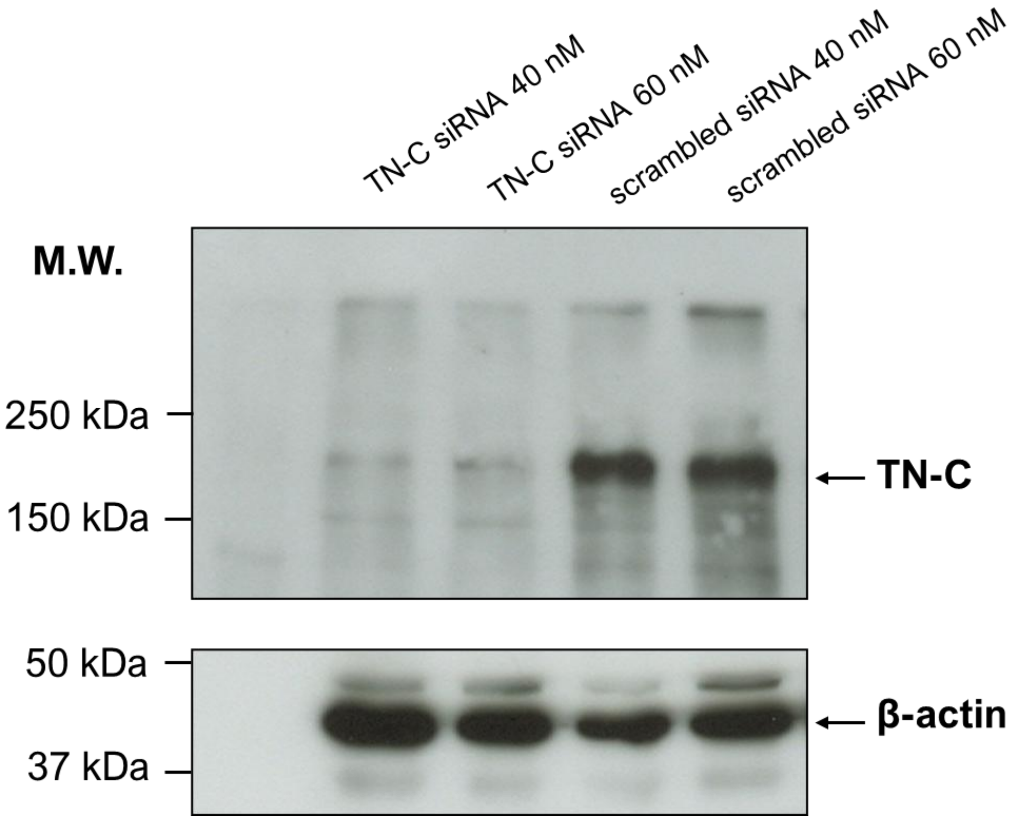

Figure S4 – Analysis of TN-C silencing in U251-MG cells by Western blotting. TN-C silencing was confirmed in U251-MG cells by SDS-PAGE and immunoblotting. Cell lysates were prepared in Triton X-100 lysis buffer with protease inhibitors, resolved on 6% (TN-C) or 10% (β-actin) SDS-PAGE gels, transferred to PVDF membranes, and probed with specific antibodies, followed by ECL detection, as detailed in the Material & Methods section.

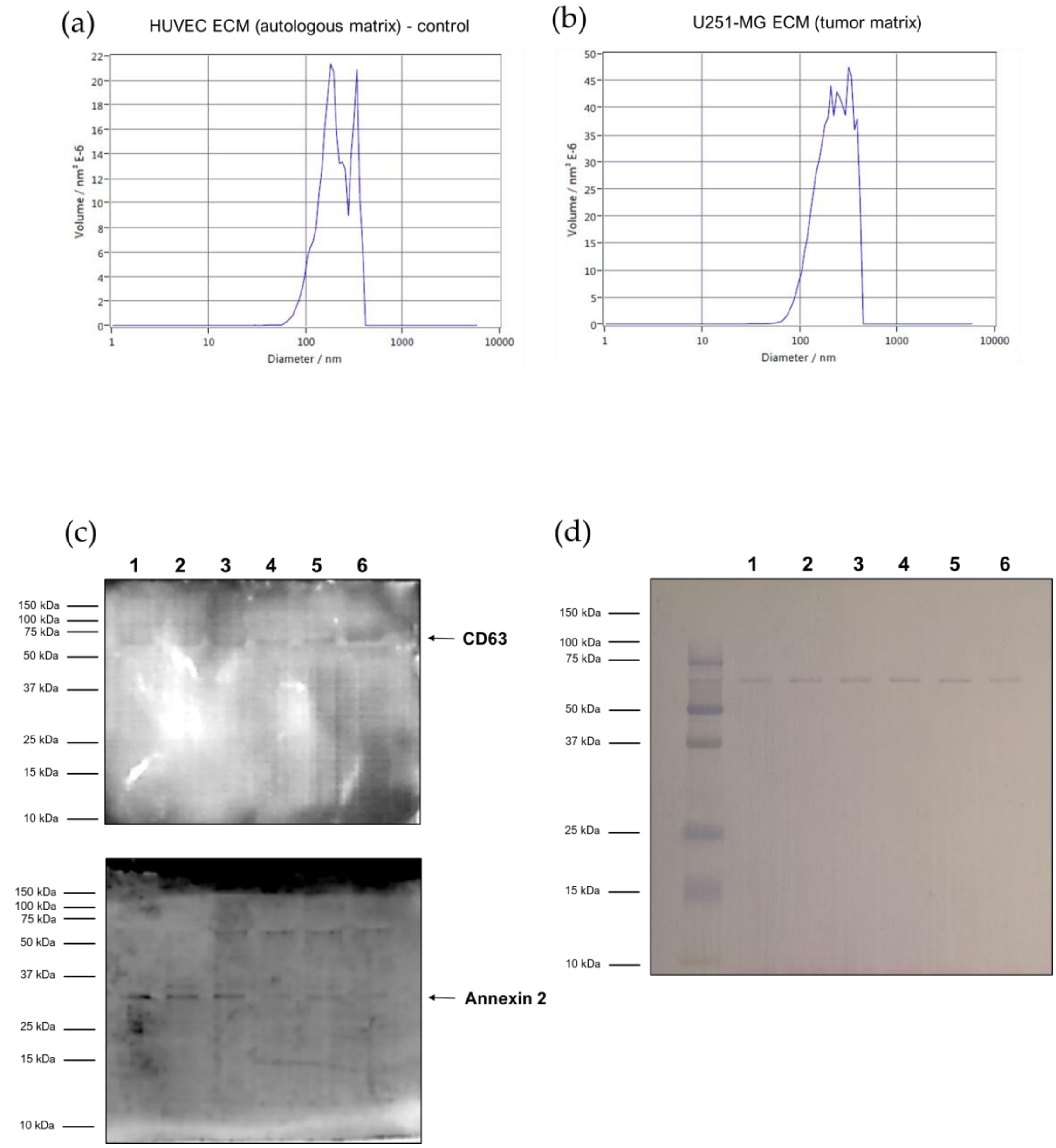

Figure S5 – Characterization of endothelial-derived extracellular vesicles (EVs). (a,b) Nanoparticle tracking analysis (NTA) of EVs isolated from (a) Evs from control conditions (HUVECs adhering to autologous extracellular matrix) and (b) Evs from TDECs (HUVECs adhering to astrocytoma matrix). EV size distribution and concentration were measured using a NanoSight instrument, as described in the Materials and Methods section. **(c,d)** EVs lysates were prepared in RIPA lysis buffer supplemented with protease inhibitors, resolved by 12% SDS-PAGE (5 μg total protein per lane), transferred to PVDF membranes, and (c) probed with the indicated antibodies, followed by ECL detection, as described in the Materials and Methods section; (d) Equal protein loading across samples was verified by PVDF membrane staining with 0.1% Coomassie Blue R-250. Lanes 1-3: three independent preparations of EVs from the autologous ECM condition (control); lanes 4-6: three independent preparations of EVs from the astrocytoma ECM condition.

(a)

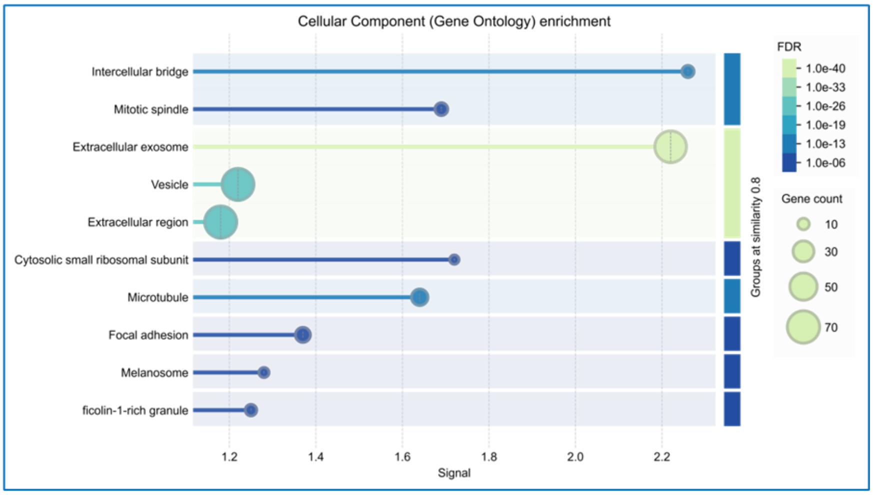

(b)

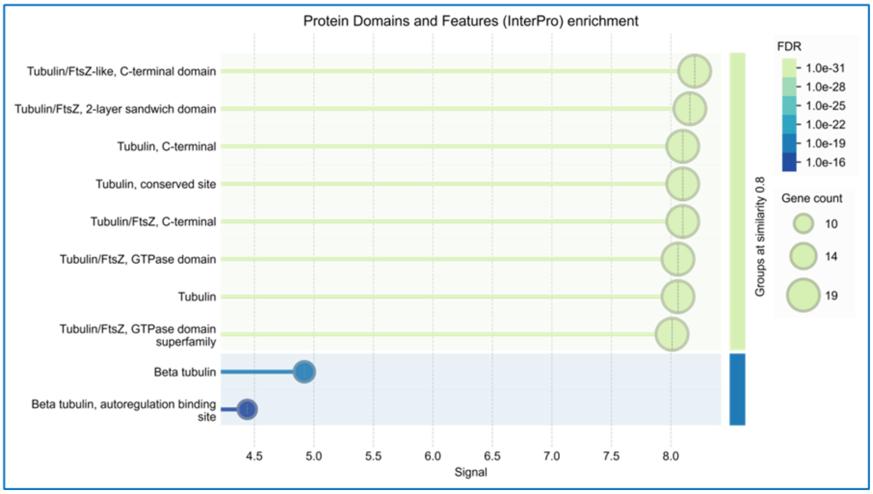

Figure S6:
Enrichment analysis of GO terms related to cellular components and protein domains and features (InterPro) in the proteome of TDECs (or U251 ECM condition). **(a)** Dot plot of upregulated proteins, highlighting strong over-representation of mitosis-related processes, including spindle organization, cytokinesis, and cell-cycle progression; **(b)** Enriched InterPro domains predominantly correspond to cytoskeletal regulators, tubulin-related modules, small GTP-binding proteins, and trafficking factors, reflecting the structural bias of the upregulated proteome toward cytoskeleton remodeling and vesicle dynamics.

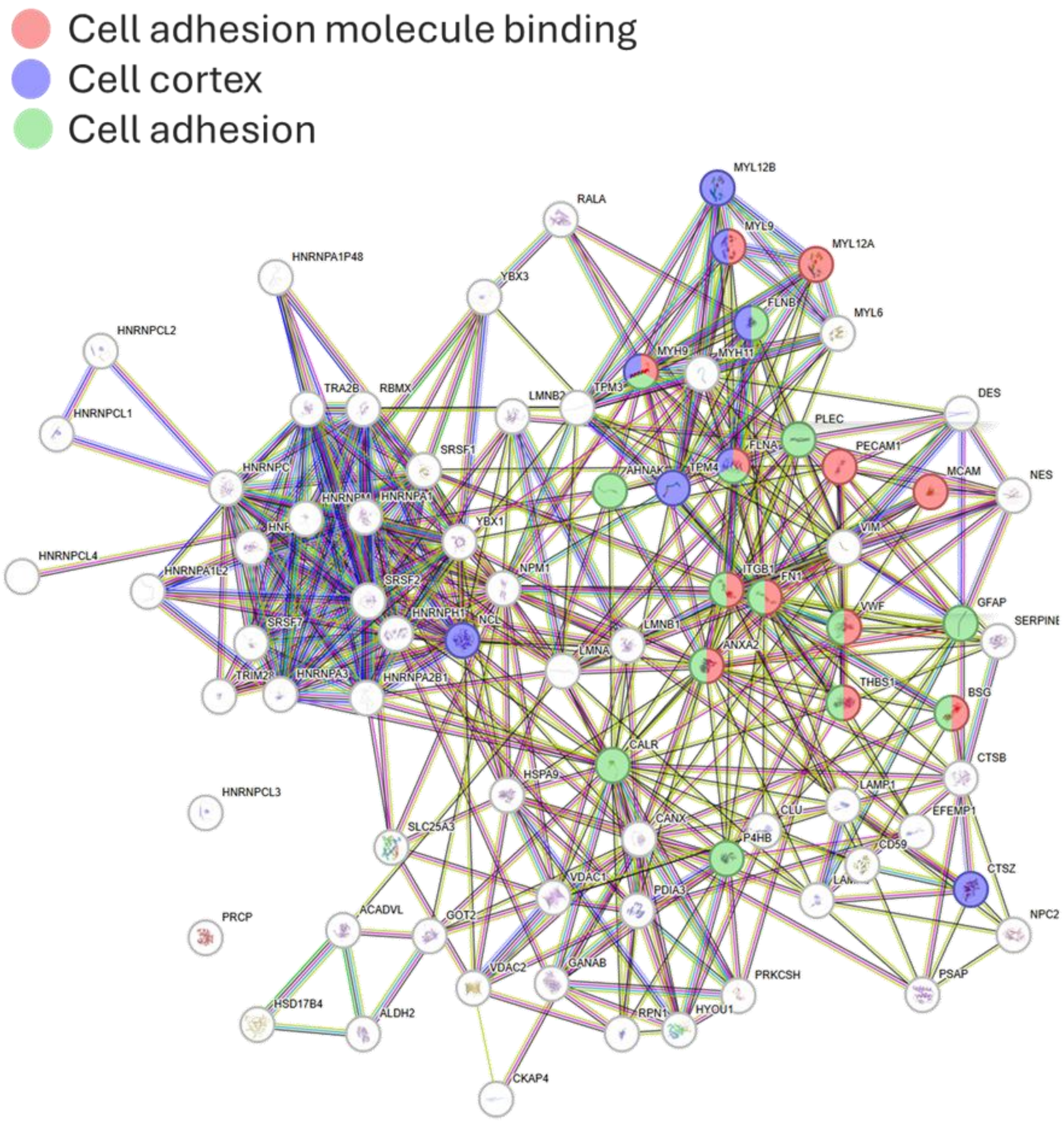

Figure S7:
STRING network analysis of regulated proteins in endothelial cells primed with autologous ECM (HUVEC ECM; control condition): a focus on cell adhesion-related nodes. Nodes annotated with “cell adhesion molecule binding” (red), “cell cortex” (blue), and “cell adhesion” (green) outline a cohesive interaction landscape characteristic of a stable, adhesion-competent endothelial phenotype.
